# Vector competence of *Aedes albopictus* and *Aedes aegypti* from the islands of the Southwestern Indian Ocean for epidemic Zika, dengue, and chikungunya viruses

**DOI:** 10.1186/s13071-025-07193-0

**Published:** 2025-12-12

**Authors:** Sarah Hafsia, Yann Gomard, Clément De Graaf, Fiona Baudino, Haoues Alout, Cyrille Lebon, Patrick Rabarison, Ambdoul-bar Idaroussi, Amina Yssouf, Simon Julienne, David A. Wilkinson, Célestine Atyame, Patrick Mavingui

**Affiliations:** 1 Université de La Réunion, UMR PIMIT (Processus Infectieux en Milieu Insulaire Tropical), CNRS 9192/INSERM 1187/IRD 249/Université de La Réunion, Île de La Réunion, France; 2 Unité Mixte de Recherche Animal Santé Territoires Risques Ecosystèmes, CIRAD/INRAE/Université de Montpellier, Université de Montpellier, 34398 Montpellier, France; 3 Service de lutte Antivectorielle, ARS Mayotte, Centre Kinga 97600, Kawéni, Mayotte France; 4 National Malaria Control Program, Moroni, Comoros; 5 Ministry of Health, Victoria, Mahé Seychelles; 6 Present Address: Unité Mixte de Recherche Animal Santé Territoires Risques Écosystèmes, CIRAD/INRAE/Université de Montpellier, Plateforme Technologique CYROI, La Réunion, France

**Keywords:** *Aedes albopictus*, *Aedes aegypti*, Arboviruses, Vector competence, Islands of the Southwestern Indian Ocean, Transmission risk

## Abstract

**Background:**

*Aedes albopictus* and *Aedes aegypti* are key vectors involved in the transmission of human pathogens worldwide. Epidemiological studies have demonstrated varying levels of arbovirus transmission by these mosquito vectors, leading to an increasing number of investigations that assess vector competence (the ability of an insect to become infected and subsequently transmit a pathogen) of *Ae. albopictus* and *Ae. aegypti* lines, to decipher the risks associated with each species. In this study, we evaluated the vector competence of *Ae. albopictus* and *Ae. aegypti* lines from the Southwestern Indian Ocean (SWIO) for three arboviruses: Zika virus (ZIKV), dengue virus serotype-1 (DENV-1), and chikungunya virus (CHIKV).

**Methods:**

Ten mosquito lines (eight *Ae. albopictus* and two *Ae. aegypti* lines), collected from five islands within SWIO (the Seychelles, the Comoros, and the Mascarene archipelagoes), were exposed to epidemic strains of ZIKV, DENV-1, and CHIKV. Three vector competence parameters (infection rate [IR], dissemination efficiency [DE], and transmission efficiency [TE]) were assessed at different days post exposure (dpe) to infectious blood meals, using plaque forming unit (PFU) assays. In addition, viral loads were quantified in positive saliva. These parameters were then compared between mosquito lines, geographic origins, and dpe for each virus.

**Results:**

None of the mosquito lines were competent for the ZIKV strain tested. In contrast, both *Ae. albopictus* and *Ae. aegypti* lines were competent vectors for the strains of DENV-1 and CHIKV tested, with transmission efficiencies reaching 35.4% for DENV-1 and 62.5% for CHIKV. For both mosquito species, statistical analyses revealed that dpe significantly influenced vector competence parameters, whereas the geographic origin of mosquito lines did not.

**Conclusions:**

The observed vector competence patterns for the three tested viruses might partly explain their current epidemiology in the SWIO. This approach should involve a larger number of *Ae. aegypti* lines and should be extended to other ZIKV, DENV, and CHIKV strains, as well as to viruses not currently reported in the region, to better assess the risk of (re-)emergence of mosquito-borne viruses in the SWIO.

**Graphical Abstract:**

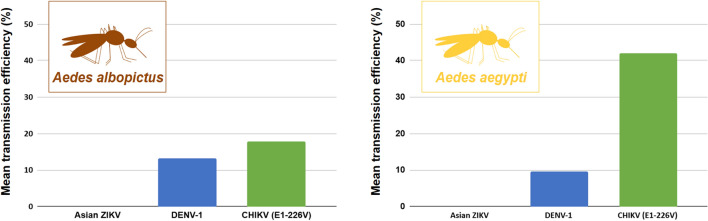

**Supplementary Information:**

The online version contains supplementary material available at 10.1186/s13071-025-07193-0.

## Background

Mosquitoes are vectors of the most important human vector-borne diseases in terms of mortality and morbidity, causing an estimated 725,000 deaths annually [1–4]. Although more than 3600 mosquito species have been described worldwide, only a few are involved in the transmission of pathogens [5]. Among these, the yellow fever mosquito *Aedes aegypti* (Linnaeus, 1762) and the Asian tiger mosquito *Aedes albopictus* (Skuse, 1894) are of major public health concern as they transmit several arthropod-borne viruses (arboviruses), including Zika virus (ZIKV) and dengue virus (DENV) that belong to family Flaviviridae as well as chikungunya virus (CHIKV, family Togaviridae) [6–10].

*Aedes aegypti* is native to Africa and occurs mainly in subtropical and tropical regions. Its global expansion beyond Africa occurred within the past 500 years [11]. In contrast, *Ae. albopictus* is native to Asia, and its spread has been more recent, accelerating in 1980s owing to international trade in used tires [7, 12, 13]. The physiological and ecological plasticity of *Ae. albopictus*, including egg diapause, opportunistic feeding behavior, and its ability to exploit both natural and artificial larval breeding sites, has facilitated its dispersal and adaptation to new environments [7, 13]. Today, *Ae. albopictus* is considered one of the most invasive species globally, present on all continents except Antarctica [13]. Owing to its frequent invasion into new areas, the geographic distribution of *Ae. albopictus* now overlaps with that of *Ae. aegypti* in several regions, including the islands of the Southwestern Indian Ocean (SWIO).

The islands of the SWIO include Madagascar, the Comoros archipelago (comprising four islands: Grande Comore, Mohéli, Anjouan, and Mayotte), the Seychelles (consisting of several coral and granite islands such as Mahé and Praslin), the Mascarenes (including Reunion Island, Mauritius, and Rodrigues), as well as numerous islets known as the Iles Eparses (Europa, Bassas da India, Juan de Nova, Grande Glorieuse, and Tromelin). On most of these islands, both *Ae. aegypti* and *Ae. albopictus* have been reported [14, 15]. However, recent entomological surveys indicate a significant reduction in the distribution of *Ae. aegypti,* particularly on Reunion Island, Rodrigues, and the granitic islands of the Seychelles, likely owing to competitive displacement by *Ae. albopictus* [15].

In recent years, the islands of the SWIO have experienced several arbovirus outbreaks in human populations with different epidemiological profiles between islands, as illustrated by outbreaks of DENV and CHIKV. For DENV, the first outbreak was documented in 1943 in the Comoros archipelago, followed by outbreaks on Reunion Island and in the Seychelles in 1977–1978 [16]. Over the subsequent four decades, DENV was detected mainly in sporadic cases, although outbreaks occurred in Madagascar (2006) and Mauritius (2008) [16]. Between 2017 and 2021, Reunion Island faced an unprecedented DENV epidemic, with 71,636 confirmed cases, 542 severe forms and 78 reported deaths, whereas few human cases were reported on other SWIO islands over the same period [16]. For CHIKV, the first wave of epidemics that hit the SWIO was reported in 2005 in the Comoros archipelago, with a high incidence on Grande Comore, while the epidemic was considerably less severe on the other Comorian islands, including Mayotte [17]. In 2006, a second larger epidemic wave affected Mayotte, Reunion Island, Mauritius, the Seychelles, and Madagascar [18]. Notably, this second wave was not reported on three other islands of the Comoros archipelago. These epidemiological contrasts are further highlighted by a serological study showing differences in human exposure to DENV, CHIKV, and West Nile virus across the three islands of the Comoros archipelago (Grande Comore, Mohéli, and Anjouan) [19]. While geographic and human-related factors may partly explain these epidemiological differences, the role of mosquito vectors in the SWIO, particularly variations in their vector competence should also be considered.

The aim of this study was to experimentally evaluate the vector competence of eight *Ae. albopictus* and two *Ae. aegypti* lines from five islands of the SWIO (Mahé and Praslin from the Seychelles archipelago; Grande Comore and Mayotte from the Comoros archipelago; and Reunion Island from the Mascarene archipelago). These mosquito lines were exposed to three arboviruses: DENV serotype 1 (DENV-1) and CHIKV, both of which are already circulating in the SWIO; and ZIKV, for which no autochthonous transmission has been reported to date in the region. The results of this study shed light on the interactions between *Ae. albopictus* and *Ae. aegypti* from the SWIO and DENV-1, CHIKV, and ZIKV, and could improve our understanding of the role of these mosquito species in shaping the epidemiological patterns of arboviruses in the region.

## Methods

### Mosquito lines

**Fig. 1:**
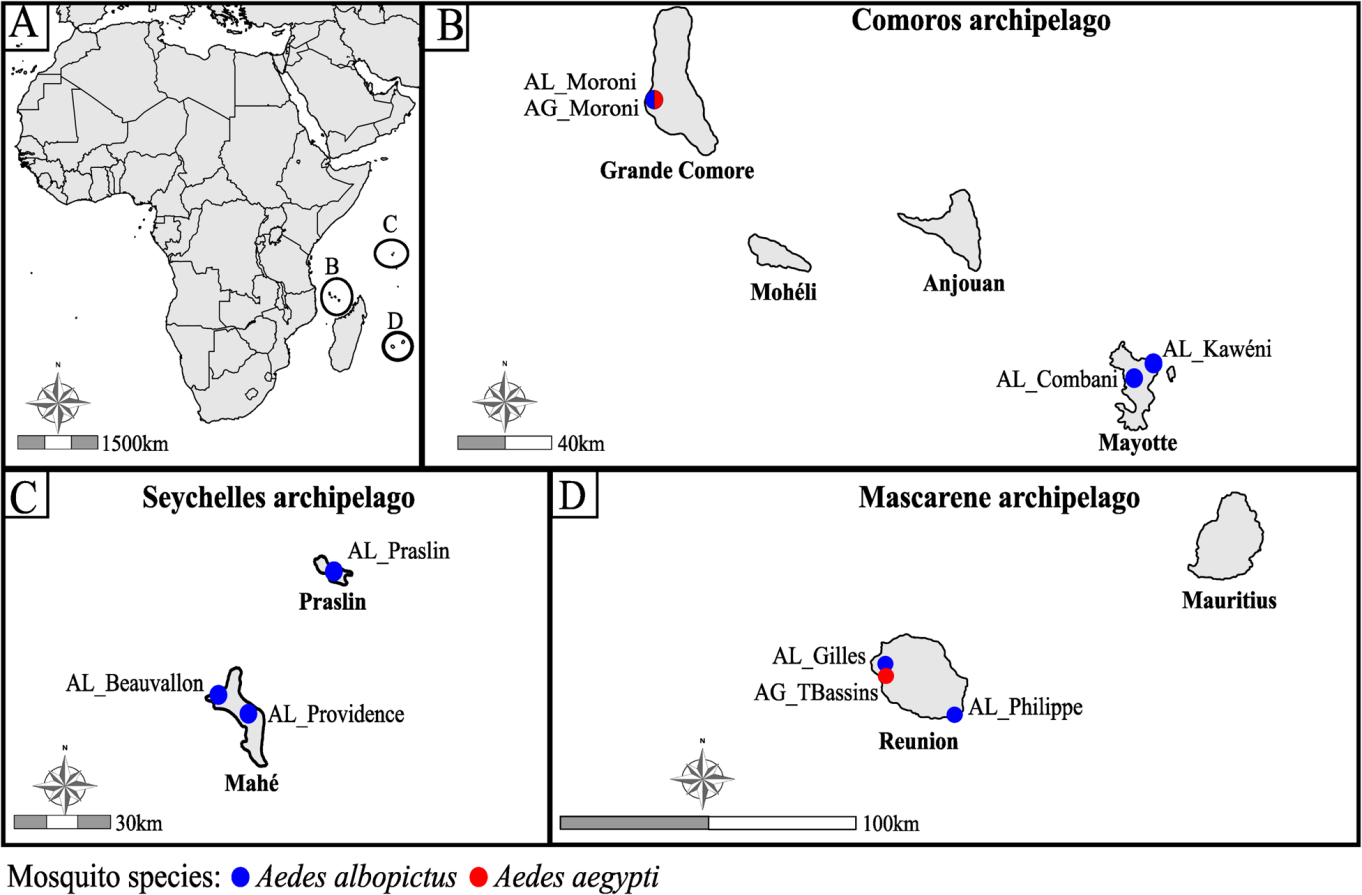
Maps showing collection sites of *Aedes albopictus* and *Aedes aegypti* mosquitoes in the SWIO**. A** Localization of surveyed islands in the SWIO. Sampling sites in the (**B**) Comoros archipelago, **C** Seychelles archipelago, and (**D**) Mascarene archipelago. Blue and red points indicate sites where *Ae. albopictus* and *Ae. aegypti* mosquitoes, respectively, were collected to establish laboratory colonies

A total of eight *Ae. albopictus* lines (AL) and two *Ae. aegypti* lines (AG), collected from islands in the SWIO in 2014 and 2019, were used (Additional File 1). Among *Ae. albopictus* lines, three originated from the Seychelles archipelago (two from Mahé: AL_Beauvallon and AL_Providence; and one from Praslin: AL_Praslin); three from the Comoros archipelago (one from Grande Comore: AL_Moroni; and two from Mayotte: AL_Combani and AL_Kaweni); and two from Reunion Island within the Mascarene archipelago (AL_Gilles and AL_Philippe). For *Ae. aegypti*, one line was collected from Grande Comore (AG_Moroni) and one from Reunion (AG_TBassin) (Fig. 1 and Additional File 1). Mosquitoes were sampled as eggs and were reared in the insect laboratory under standard conditions (27 ± 1 °C, 80% relative humidity (RH), 12 h light/12 h dark photoperiod). Larvae were fed ad libitum with yeast tablets, and adults were provided with 10% sucrose solution.

### Viruses

Three arboviruses were used: ZIKV, DENV-1, and CHIKV. The ZIKV strain corresponds to an Asian lineage (PF-251013–18; GenBank accession no.: KJ579442) isolated from a human in French Polynesia in 2013 [20]. The DENV-1 used is the PR-1583 strain of serotype 1 (GenBank accession no.: ON631277) that was isolated from human serum in Reunion in 2019 [21]. The CHIKV strain (UVE/CHIKV/2006/RE/LR2006_OPY1; GenBank accession no.: DQ443544), isolated from a human in Reunion Island in 2006, carries the E1-226 V mutation in the E1 structural protein, which is associated with increased virus replication and transmission in *Ae. albopictus* [6]. The CHIKV strain was provided as a lyophilized preparation (eighth passage) by the European Virus Archive goes global (EVAg). The three arboviruses were amplified in Vero E6 cells (ATCC, ref. no. CRL-1586) at a multiplicity of infection (MOI) of 0.1 in an Eagle’s minimum essential medium (MEM) supplemented with 2% heat-inactivated fetal bovine serum (FBS), 2 mmol/l l-glutamine, 1 mmol/l sodium pyruvate, 10 U/ml penicillin, 0.1 mg/ml streptomycin and 0.5 μg/ml fungizone (PAN Biotech, Aidenbach, Germany). To produce virus stocks, Vero cells were maintained at 37 °C with a 5% CO_2_ atmosphere. Supernatants were harvested 2 days (for CHIKV) and 5 days (for ZIKV and DENV-1) post infection, and stored at −80 °C until experimental infections. The ZIKV, DENV-1, and CHIKV used were at their sixth, fourth, and tenth passages, respectively.

### Experimental infections of mosquitoes

Experimental infections were performed with *Ae. albopictus* lines from generations F_1_–F_9_ and *Ae. aegypti* lines from generations F_2_–F_6_ for the AG_Moroni line and F_31_–F_37_ for the AG_Tbassin line (Additional File 1). Female mosquitoes aged 10 days were placed in plastic boxes within a climatic chamber and starved for 48 h under standard conditions (27 ± 1 °C; 80% RH; 12 h light/12 h dark). Then, mosquitoes were offered a blood meal consisting of 1.4 ml washed rabbit erythrocytes, 700 μl virus (either ZIKV, DENV-1 or CHIKV), and 5 mM (21 μl) adenosine triphosphate (ATP) as phagostimulant. The final viral titers in the infectious blood meals were 10^6^ PFU/ml for ZIKV and DENV-1 and 10^8^ PFU/ml for CHIKV. These viral titers are consistent with those used in previous studies [10, 22–24]. Blood meals were provided using a Hemotek feeding system (Hemotek Limited, Great Harwood, UK) with pig intestines serving as membranes. Female mosquitoes were allowed to feed for between 30 min and 1 h. Only fully engorged females were subsequently transferred to a climatic chamber and maintained with 10% sucrose up to 21 days for ZIKV, 28 days for DENV-1 and 14 days for CHIKV. Given the scope of the study and the large number of samples, only one replicate was performed for each mosquito line and virus combination.

### Mosquito processing

A forced salivation method [25] was used to collect saliva from individual mosquitoes (19–48 specimens) at 7, 14, and 21 days post exposure (dpe) for ZIKV; at 14, 21, and 28 dpe for DENV-1; or at 7 and 14 dpe for CHIKV (Additional File 1). For each mosquito, the legs and wings were removed before introducing the proboscis into a 20 μl pipette tip containing 5 μl FBS to collect the saliva. After 45 min (for ZIKV and CHIKV) or 30 min (for DENV-1), the solution containing saliva was mixed with 45 μl MEM supplemented with 2 mmol/l l-glutamine, 1 mmol/l sodium pyruvate, 10 U/ml penicillin, 0.1 mg/ml streptomycin, and 0.5 μg/ml fungizone. The head, thorax, and abdomen of each specimen were then grounded separately in 200 μl MEM medium supplemented with 2% FBS and centrifuged at 10,000*g* for 5 min to pellet tissue debris. Finally, 150 μl of the supernatant of each sample was stored at −80 °C until virus detection and titration.

### Virus detection, titration, and evaluation of vector competence

The viral infection status of mosquito tissues and saliva was determined using plaque forming unit (PFU) assays. To detect ZIKV and DENV-1, Vero E6 cells were seeded either in 48-well culture plates at a density of 4 × 10^4^ cells per well for testing mosquito tissue homogenates or in six-well culture plates at a density of 5 × 10^5^ per well for saliva samples. The following day, cells were infected with tenfold dilutions of mosquito tissue homogenates or saliva. For the detection of infectious CHIKV particles, Vero E6 cells were seeded in 96-well culture plates at a density of 2 × 10^4^ cells per well or in 12-well culture plates at a density of 3 × 10^5^ cells and incubated at 37 °C with a 5% CO_2_ atmosphere. The next day, cells in 96-well culture plates were infected with tenfold dilutions of mosquito tissue homogenates, while cells in 12-well culture plates were infected with tenfold dilutions of saliva. After infection with each of the three viruses, MEM medium supplemented with 5% of FBS, 2 mmol/l l-glutamine, 1 mmol/l sodium pyruvate, 10 U/ml penicillin, 0.1 mg/ml streptomycin, 0.5 μg/ml fungizone, and 0.8% carboxymethylcellulose sodium salt (CMC; Sigma-Aldrich, Saint-Quentin-Fallavier, France) was added to each well. The cells were then incubated at 37 °C with a 5% CO_2_ atmosphere for 3, 5 and 7 days for CHIKV, DENV-1, and ZIKV, respectively. Subsequently, the medium was removed, and a solution of 3.7% paraformaldehyde (Sigma-Aldrich) was used to fix cells. Finally, the cells were stained with 0.5% crystal violet (Sigma-Aldrich) diluted in 20% ethanol and the presence or absence of infectious viral particles in the samples was assessed. In addition, the number of PFU were quantified in the saliva samples.

**Fig. 2:**
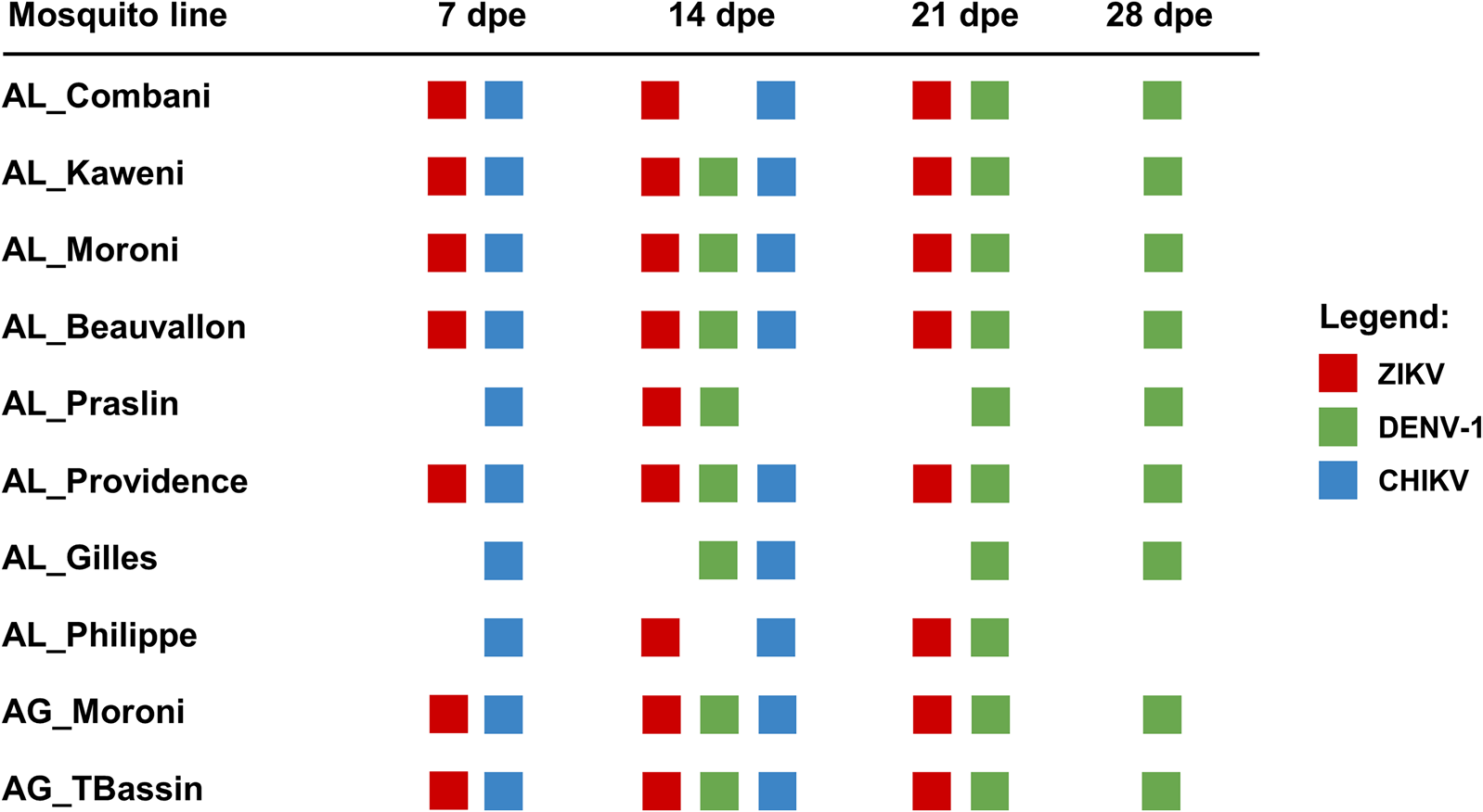
Virus testing schedule across days post exposure (dpe) for each mosquito line. Rows represent mosquito lines, columns correspond to time points (7, 14, 21, and 28 dpe), and colored squares denote tested viruses: ZIKV (red), DENV-1 (green), and CHIKV (blue)

Vector competence was evaluated on the basis of three parameters: (i) the infection rate (IR), (ii) the dissemination efficiency (DE), and (iii) the transmission efficiency (TE) in line with previous studies [10, 22–24]. The IR corresponded to the proportion of infected bodies (abdomen and thorax) among examined mosquitoes, the DE was defined as the proportion of mosquitoes with infected heads among examined mosquitoes, and the TE corresponded to the proportion of females with virus in the saliva among examined mosquitoes [10]. Regarding the exposure of the eight *Ae. albopictus* lines to each of the three viruses: seven lines were exposed to ZIKV, with analyses conducted at 7, 14, and 21 dpe for five lines; at 14 and 21 dpe for one line; and at 14 dpe for one line. For DENV-1, all eight lines were exposed, with analyses performed at 14, 21, and 28 dpe for six lines; at 21 and 28 dpe for one line; and at 21 dpe for one line. For CHIKV, among the eight lines, analyses were conducted at 7 and 14 dpe for seven lines and at 7 dpe for one line (Fig. 2). For the two *Ae. aegypti* lines exposed to infectious blood meals, analyses were performed at 7, 14, and 21 dpe for ZIKV; at 14, 21, and 28 dpe for DENV-1; and at 7 and 14 dpe for CHIKV (Fig. 2).

### Statistical analyses

Statistical analyses were performed separately for *Ae. albopictus* and *Ae. aegypti* owing to an unbalanced number of mosquito lines between the species (eight lines versus two lines). The response variable of vector competence (IR, DE, and TE) for each virus used was analyzed separately using generalized linear models (GLMs) with a binomial error distribution. For each GLM, the explanatory variables tested included mosquito line (categorical variable with eight levels for *Ae. albopictus* and two for *Ae. aegypti*), dpe (numerical variable), and geographical region (categorical variable with three levels: i.e., Comoros archipelago, Seychelles archipelago, and Reunion Island), as well as their interactions. For *Ae. aegypti*, to avoid confounding effects between mosquito lines and geographical regions, only mosquito lines, dpe, and their interactions were examined. The selection of the best models was performed with the function ‘step’ from R software version 3.6.2 [26]. Then the significance of each variable was assessed using the ‘Anova’ function from the ‘car’ R package, which performs a type III hypothesis test [27]. On the basis of the best GLM, the ‘emmeans’ R package [28] was used to assess the statistical differences and to compute the estimated marginal means (‘emmeans’), which are presented as examples to illustrate these statistical differences in the Results section. Among the three explanatory variables, the geographical region variable was not retained in any of the best models and did not influence the observed vector competence parameters for each virus.

For each virus and mosquito species, the viral loads in positive saliva were compared between the mosquito lines for each dpe and between the dpe within each mosquito line using nonparametric tests of Mann–Whitney (for comparisons between two groups) or of Kruskal–Wallis followed by a post hoc Dunn test (for multiple comparisons between more than two groups). When significant, differences between saliva viral loads were illustrated presenting medians to be closer to the nonparametric test’s comparisons. The tests were conducted only when the number of positive samples in mosquito line at a specific dpe was ≥ 3. Hence, data at 14 dpe were removed from the statistical analyses for DENV-1 because of a too low number of positive saliva (*N* = 2). All statistical analyses were performed using the R software.

## Results

### *Aedes albopictus* and *Ae. aegypti* lines are not competent vectors for the ZIKV strain

**Fig. 3:**
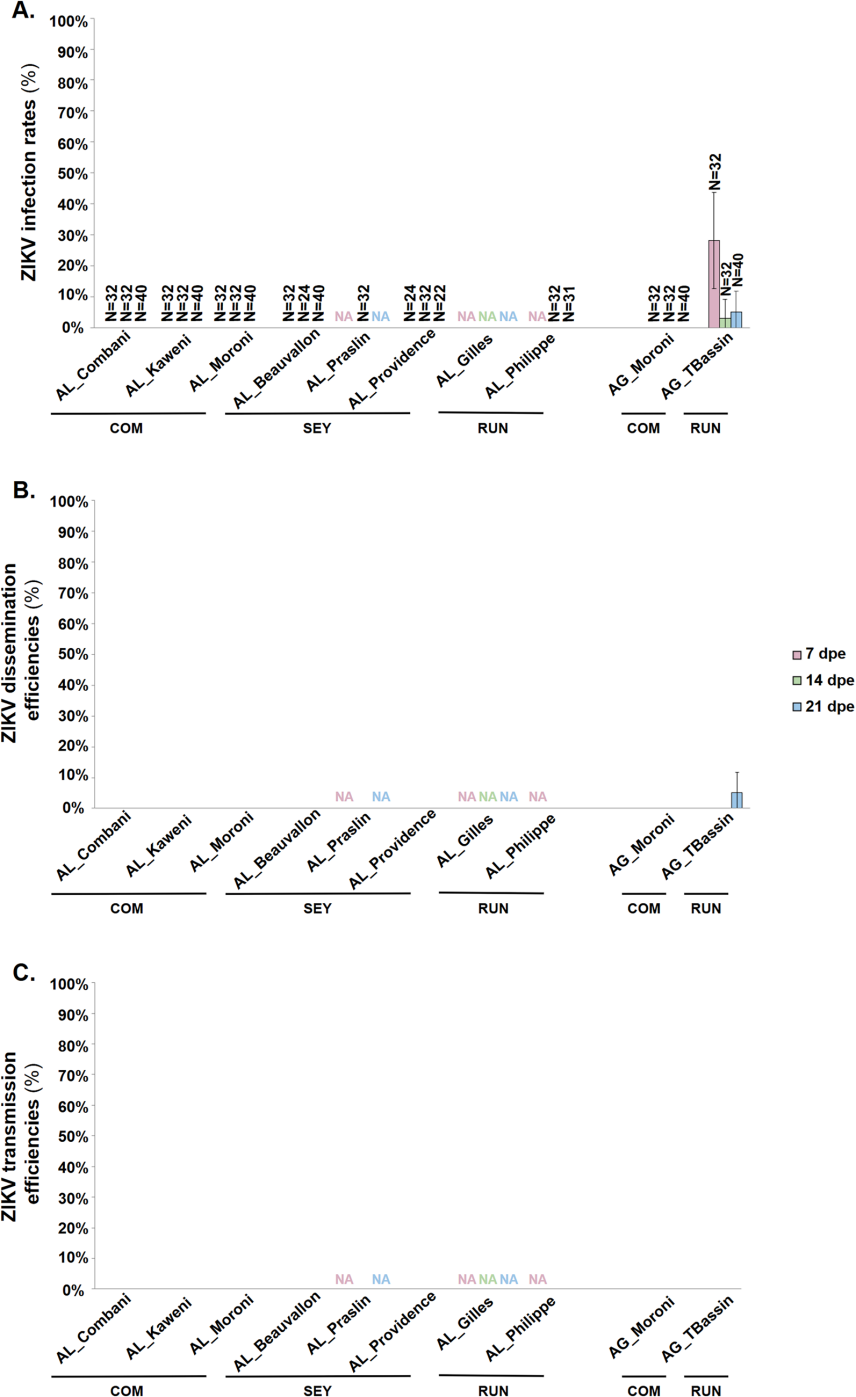
Vector competence parameters of *Aedes albopictus* and *Aedes aegypti* lines exposed to the ZIKV strain. Graphics A–C correspond to infection rate (IR), dissemination efficiency (DE), and transmission efficiency (TE), respectively. Mosquito lines were tested at 7, 14, and 21 days post exposure (dpe) to the ZIKV. Samples were examined for the presence of infectious viral particles by plaque forming unit (PFU) titration on Vero cells: bodies for the IR, heads for the DE, and saliva for the TE. Error bars correspond to the 95% confidence interval. *COM* Comoros archipelago, *SEY* Seychelles archipelago, *RUN* Reunion Island, *N*, number of mosquitoes tested, *NA* not available

Exposure of mosquito lines to the Asian ZIKV strain PF-251013–18 did not result in the detection of infectious ZIKV particles in the body, head, or saliva of any of the *Ae. albopictus* lines tested at any dpe (Fig. 3 and Additional File 2). The same nonpermissiveness to ZIKV was observed in the *Ae. aegypti* line from Grande Comore (AG_Moroni). In contrast, the line from Reunion Island (AG_TBassin) showed body infection at 7 dpe (IR = 28.1%; 15.6–45.4%), 14 dpe (IR = 3.1%; 0.6–15.7%), and 21 dpe (IR = 5.0%; 1.4–16.5%), while disseminated ZIKV infection was detected only at 21 dpe (DE = 5.0%; 1.4–16.5%), with no evidence of transmission (Fig. 3 and Additional File 2).

### *Aedes albopictus* and *Ae. aegypti* lines are competent vectors for the DENV-1 strain

**Fig. 4:**
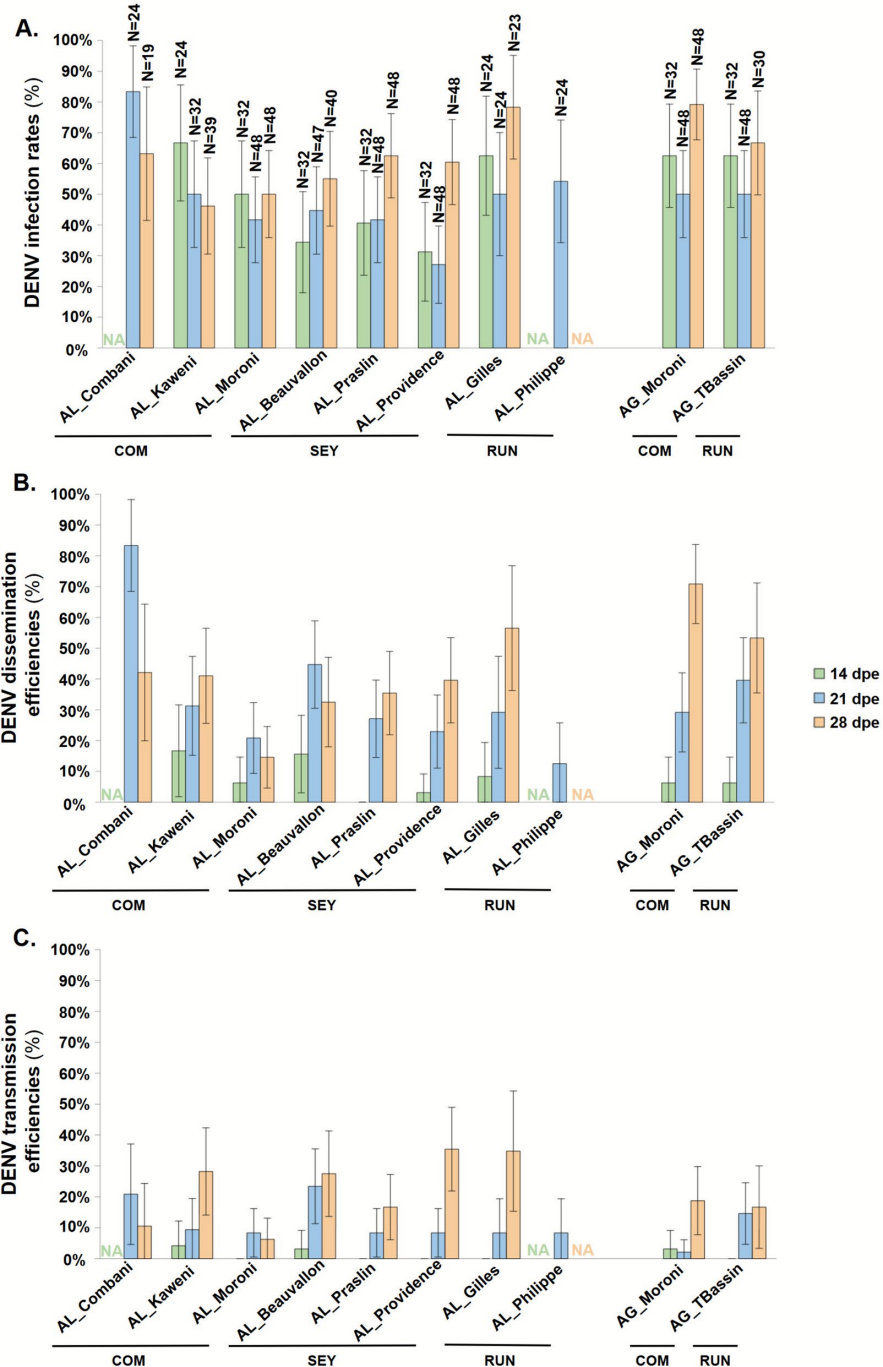
Vector competence parameters of *Aedes albopictus* and *Aedes aegypti* lines exposed to the DENV-1 strain. Graphics (**A**–**C**) correspond to infection rate (IR), dissemination efficiency (DE), and transmission efficiency (TE), respectively. Mosquito lines were tested at 14, 21, and 28 days post exposure (dpe) to DENV-1. Samples were examined for infectious viral particles plaque forming unit (PFU) titration on Vero cells: in the bodies for the IR, the heads for DE, and the saliva for TE. Error bars represent 95% confidence intervals. *COM* Comoros archipelago, *SEY* Seychelles archipelago, *RUN* Reunion Island, *N* number of mosquitoes tested, *NA* not available

All tested *Ae. albopictus* lines were competent for the DENV-1 strain (Fig. 4 and Additional File 3). The IR was significantly influenced by dpe (GLM,* X*^*2*^ = 10.27, *df* = 2, *P*-value = 0.006) and by mosquito line (GLM, *X*^*2*^ = 23.03, *df* = 7, *P*-value = 0.002) (Fig. 4A and Table 1). The effect of mosquito line was notably driven by the significant difference between the highest mean IR of AL_Combani (74.3%; 59.0–85.4%) and the lowest of AL_Providence (40.2%; 32.0–49.0%, *P*-value = 0.006, Additional File 4). Disseminated DENV-1 infection was observed in all lines and all dpe, except at 14 dpe for AL_Praslin (Fig. 4B and Additional File 3). The DE was significantly influenced by the dpe (GLM, *X*^*2*^ = 48.53, *df* = 2, *P*-value < 0.001), the mosquito line (GLM, *X*^*2*^ = 41.20, *df* = 7, *P*-value < 0.001), and the interaction between the dpe and mosquito line (GLM, *X*^*2*^ = 26.49, *df* = 11, *P*-value = 0.005, Table 1). Indeed, the DE of the different mosquito lines evolved differently over time (Fig. 4B; Additional File 4). All mosquito lines tested presented females with infectious DENV-1 particles in saliva at 21 and 28 dpe, and two lines (AL_Kaweni and AL_Beauvallon) even showed females with positive saliva as early as 14 dpe (Fig. 2C and Additional File 3). The TE was significantly influenced by dpe (GLM, *X*^*2*^ = 54.94, *df* = 2, *P*-value < 0.001, Table 1) and increased significantly over time (Fig. 4C; Additional File 4). TE was also influenced by mosquito line (*X*^*2*^ = 17.6, *df* = 7, *P*-value = 0.014, Table 1), with the effect being driven by the significantly higher mean TE of AL_Beauvallon (11.5%; 6.4–19.8%) compared with the lowest, observed in AL_Moroni (2.7%; 1.1–6.3%) (*P*-value = 0.017, Additional File 4).

**Table 1:** Statistical analysis of vector competence parameters after exposition to the DENV-1 strain. The effects of mosquito lines (AL_Combani, AL_Kaweni, AL_Moroni, AL_Beauvallon, AL_Praslin, AL_Providence, AL_Gilles, AL_Philippe, or AG_Moroni, AG_TBassin) and days post exposure (14, 21, or 28 dpe) were analyzed for each mosquito species separately (*Ae. albopictus* or *Ae. aegypti*). The significance of variables was determined using a GLM on the basis of the selected minimal model

Mosquito species	Source	Infection rate			Dissemination efficiency			Transmission efficiency		
		*X*^2^	*df*	*P*-value	*X*^2^	*df*	*P*-value	*X*^*2*^	*df*	*P*-value
*Aedes albopictus*	ML	23.029	7	0.002^*^	41.201	7	< 0.001^*^	17.597	7	0.014^*^
	dpe	10.272	2	0.006^*^	48.532	2	< 0.001^*^	54.944	2	< 0.001^*^
	ML × dpe	–	–	–	26.487	11	0.005^*^	–	–	–
*Aedes aegypti*	ML	–	–	–	–	–	–	0.926	1	0.336
	dpe	11.008	2	0.004^*^	57.198	2	< 0.001^*^	12.967	2	0.002^*^
	ML × dpe	–	–	–	–	–	–	6.002	2	0.049^*^

*ML* mosquito lines, *dpe* days post exposure

*Variables with a statistically significant effect; – denotes variables not included in the final model (on the basis of Akaike information criterion (AIC) selection)

The viral load of DENV-1 in positive saliva remained stable over time for each *Ae. albopictus* mosquito line, with medians ranging from 2.18 (interquartile range [IQR] = 1.94–3.60) to 3.18 (2.97–3.57) log_10_ PFU/saliva at 21 dpe and from 1.40 (1.40–1.57) to 3.10 (2.73–3.31) log_10_ PFU/saliva at 28 dpe (all Mann–Whitney test *P*-values > 0.148, Additional File 3). At 28 dpe, the viral load in positive saliva was significantly influenced by mosquito line (Kruskal–Wallis, *X*^*2*^ = 25.30, *df* = 5, *P*-value < 0.001), with AL_Beauvallon exhibiting a significantly higher median value (3.10 log_10_ PFU/saliva, IQR = [2.73–3.31]) compared with AL_Moroni, AL_Gilles, and AL_Praslin, which ranged from 1.40 (1.40–1.57) to 1.70 (1.40–2.21) log_10_ PFU/saliva (Dunn test, *P*-values ≤ 0.017, Additional File 3).

The two *Ae. aegypti* tested lines were equally competent for DENV-1 strain (Table 1 and Additional File 3). However, dpe significantly affected IR (GLM, *X*^*2*^ = 11.01, *df* = 2, *P*-value = 0.004) and the DE (GLM, *X*^*2*^ = 57.2, *df* = 2, *P*-value < 0.001, Table 1). The mean IR increased from 50.0% (40.1–59.9%) at 21 dpe to 74.4% (63.6–82.8%) at 28 dpe (*P*-value = 0.004), while the mean DE rose from 6.3% (2.4–15.5%) at 14 dpe to 34.4% (25.6–44.4%) at 21 dpe and 64.1% (53.0–73.9%) at 28 dpe (all *P*-value < 0.001, Fig. 2 and Additional File 4). The TE was also significantly influenced by dpe (GLM, *X*^*2*^ = 12.97, *df* = 2, *P*-value = 0.002) and by the interaction between dpe and mosquito line (GLM; *X*^*2*^ = 6.00, *df* = 2, *P*-value = 0.0497), with TE evolving differently over time in the two mosquito lines (Fig. 4C).

Regarding the viral loads in the positive saliva of *Ae. aegypti* mosquitoes, statistical analyses at 28 dpe (given the low number of samples for AG_Moroni at 21 dpe; Additional File 3) revealed no significant difference between the two lines (Mann–Whitney test, *P*-value = 0.228). Median viral loads were 2.24 (1.40–2.40) and 2.65 (2.00–3.23) log_10_ PFU/saliva for AG_TBassin and AG_Moroni, respectively (Additional File 3).

### *Aedes albopictus* and *Ae. aegypti* lines are competent vector for the CHIKV strain

**Fig. 5:**
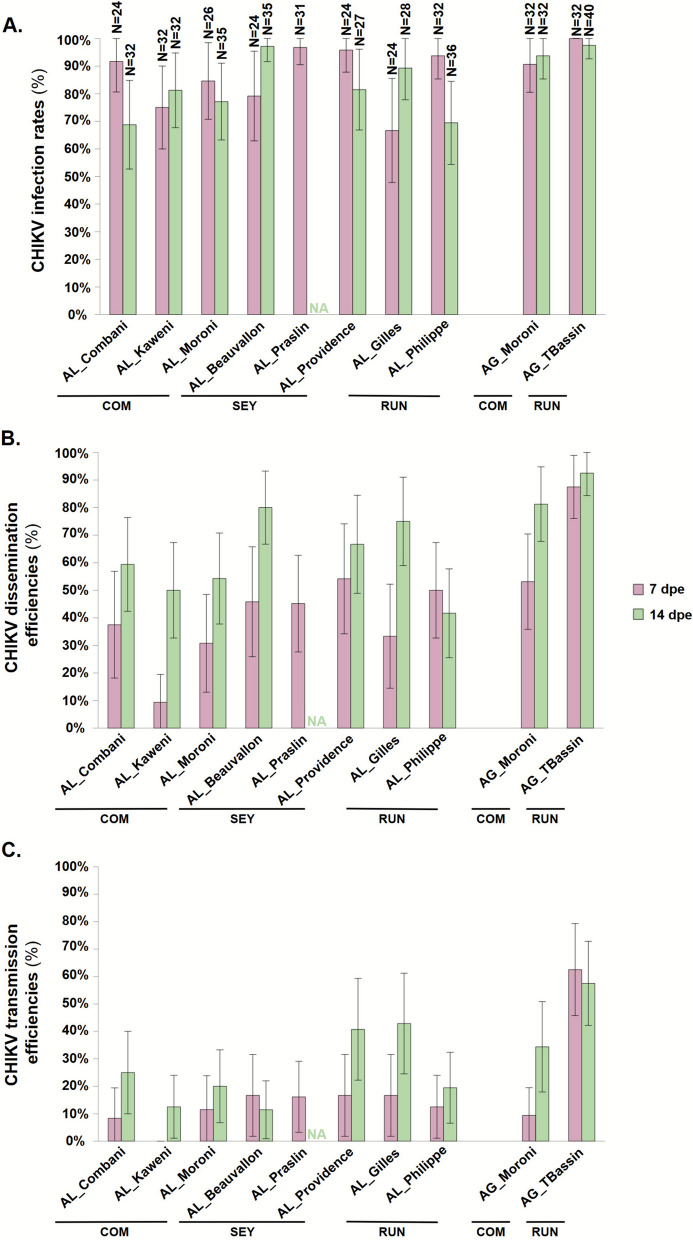
Vector competence parameters of *Aedes albopictus* and *Aedes aegypti* lines exposed to the CHIKV strain. Graphics (**A**–**C**) correspond to infection rate (IR), dissemination efficiency (DE), and transmission efficiency (TE), respectively. Mosquito lines were tested at 7 and 14 days post exposure (dpe) with the CHIKV. Samples were examined for the presence of infectious viral particles by plaque forming unit (PFU) titration on Vero cells: in the bodies for the IR, head for the DE, and saliva for the TE. Error bars correspond to the 95% confidence interval. *COM* Comoros archipelago, *SEY* Seychelles archipelago, *RUN* Reunion Island, *N* number of mosquitoes tested, *NA* not available

All tested *Ae. albopictus* lines were competent for the CHIKV strain assessed and showed highly susceptibility to CHIKV infection, with IR ranging from 66.7% at 7 dpe in AL_Gilles (46.7–82.0%) to 97.1% at 14 dpe in AL_Beauvallon (85.5–99.5%, Fig. 5A and Additional File 5). The minimal model indicated that IR was significantly influenced by the interaction between mosquito lines and dpe (GLM, *X*^*2*^ = 23.67, *df* = 6, *P*-value < 0.001, Table 2). Indeed, patterns of IR over time were different between mosquito lines (Fig. 5A). A significant change between 7 and 14 dpe was noted only in AL_Philippe with IR decreasing from 93.8% at 7 dpe (78.2–98.4%) to 69.4% at 14 dpe (52.8–82.2%, pairwise comparison *P*-value = 0.021; Additional File 6). DE was significantly affected by dpe (GLM, *X*^*2*^ = 23.04, *df* = 1, *P*-value < 0.001), mosquito line (GLM, *X*^*2*^ = 21.89, *df* = 7, *P*-value = 0.003), and the interaction between mosquito line and dpe (GLM, *X*^*2*^ = 14.67, *df* = 6, *P*-value = 0.023, Table 2). DE also varied across mosquito lines over time (Fig. 5B). Some lines showed stable DE between 7 and 14 dpe (AL_Philippe, AL_Providence, AL_Moroni, AL_Combani, all *P*-values > 0.070) while others exhibited a significant increase (AL_Kaweni, AL_Beauvallon, AL_Gilles, all *P*-values < 0.008, Additional File 5). Consequently, mosquito lines with higher DE at 7 dpe are not necessarily the same at 14 dpe (Fig. 5B, Additional File 5). TE was significantly influenced by dpe (GLM, *X*^*2*^ = 11.14, *df* = 1, *P*-value < 0.001) and mosquito line (GLM, *X*^*2*^ = 18.58, *df* = 7, *P*-value = 0.010). Mean TE increased from 11.0% (7.5–16.1%) at 7 dpe to 23.8% (18.0–30.7%) at 14 dpe (*P*-value = 0.001). AL_Kaweni showed the lowest mean TE (5.7%, 2.1–14.5%), while AL_Providence and AL_Gilles had the highest (28.0% [17.3–41.9%], *P*-value = 0.047 and 29.2% [18.3–43.0%], *P*-value = 0.033, respectively).

**Table 2:** Statistical analysis of vector competence parameters after exposition to the CHIKV strain. The effects of mosquito lines (AL_Combani, AL_Kaweni, AL_Moroni, AL_Beauvallon, AL_Praslin, AL_Providence, AL_Gilles, AL_Philippe or AG_Moroni, AG_TBassin) and days post exposure (7 or 14 dpe) were analyzed for each mosquito species separately (*Ae. albopictus* or *Ae. aegypti*). The significance of variables was determined using a GLM on the basis of the selected minimal model

Mosquito species	Source	Infection rate			Dissemination efficiency			Transmission efficiency		
		*X*^2^	*df*	*P*-value	*X*^2^	*df*	*P*-value	*X*^*2*^	*df*	*P*-value
*Aedes albopictus*	ML	10.787	7	0.148	21.889	7	0.003^*^	18.576	7	0.010^*^
	dpe	0.939	1	0.332	23.042	1	< 0.001^*^	11.141	1	< 0.001^*^
	ML × dpe	23.670	6	< 0.001^*^	14.669	6	0.023^*^	–	–	–
*Aedes aegypti*	ML	3.549	1	0.060	10.952	1	< 0.001^*^	20.250	1	< 0.001^*^
	dpe	–	–	–	5.779	1	0.016^*^	1.382	1	0.240
	ML × dpe	–	–	–	–	–	–	4.949	1	0.026^*^

*ML* mosquito lines, *dpe* days post exposure

*Variables with a statistically significant effect; – denotes variables not included in the final model (on the basis of AIC selection)

The median viral load in positive saliva samples from *Ae. albopictus* lines ranged from 2.10 (1.88–2.18) to 3.65 (3.00–3.95) log_10_ PFU/saliva at 7 dpe and from 1.80 (1.40–2.26) to 2.80 (2.35–2.88) log_10_ PFU/saliva at 14 dpe. The viral load was constant over time in AL_Beauvallon, AL_Moroni, and AL_Providence (Mann–Whitney tests, all *P*-values ≥ 0.080) but decreased significantly between 7 and 14 dpe for AL_Gilles and AL_Philippe (Mann–Whitney tests, *P*-values = 0.033 and 0.042, respectively, Additional File 5). Pairwise comparisons of viral loads between mosquito lines revealed no significant difference at either 7 dpe (Kruskal–Wallis test, *X*^*2*^ = 12.07, *df* = 5, *P*-value = 0.034; and all Dunn tests *P*-values > 0.09) or 14 dpe (Kruskal–Wallis test, *X*^*2*^ = 6.58, *df* = 6, *P*-value = 0.361).

The two *Ae. aegypti* lines tested were also competent for the CHIKV strain (Fig. 5). IR exceeded 90.0% at both time points and in both mosquito lines (Fig. 5A and Additional File 5) and were unaffected by any of the variables tested (Table 2). DE was significantly influenced by dpe (GLM, *X*^*2*^ = 5.78, *df* = 1, *P*-value = 0.016), with a higher mean DE at 14 dpe (88.8%; 79.0–94.4%) compared with 7 dpe (72.7%; 59.7–82.8%, *P*-value = 0.020; Additional File 6). Mosquito line also significantly affected DE (GLM, *X*^*2*^ = 10.95, *df* = 1, *P*-value < 0.001); the mean DE in AG_TBassin (90.8%; 81.5–95.6%) was higher than in AG_Moroni (68.3%; 55.5–78.8%, *P*-value = 0.002, Additional File 6). TE was significantly influenced by mosquito line (GLM, *X*^*2*^ = 20.25, *df* = 1, *P*-value < 0.001) and by the interaction between mosquito line and dpe (GLM, *X*^*2*^ = 4.95, *df* = 1, *P*-value = 0.026, Table 2). Notably, TE evolved differently over time in the two lines: TE in AG_Moroni increased from 9.4% (3.1–25.4%) at 7 dpe to 34.4% (20.2–52.1%) at 14 dpe (*P*-value = 0.023), while TE in AG_TBassin of 62.5% (44.9–77.3%) was higher than that of AG_Moroni at 7 dpe (*P*-value < 0.001) and remained stable over time.

The viral load in positive saliva showed a significant decrease over time for both *Ae. aegypti* lines, with medians decreasing from 3.78 (3.60–4.13) log_10_ PFU/saliva at 7 dpe to 1.70 (1.40–2 0.14) log_10_ PFU/saliva at 14 dpe for AG_Moroni (Mann–Whitney test, *P*-value = 0.012) and from 3.57 (3.30–3.81) log_10_ PFU/saliva to 2.54 (2.14–2.82) log_10_ PFU/saliva for AG_TBassin (Mann–Whitney test, *P*-value < 0.001) (Additional File 5). At 14 dpe, the viral load in positive saliva from AG_TBassin was significantly higher than that from AG_Moroni at 14 dpe (Mann–Whitney test, *P*-value = 0.001) (Additional File 5).

## Discussion

In this study, we examined the vector competence of eight *Ae. albopictus* and two *Ae. aegypti* lines from five islands in the SWIO region (Grande Comore, Mayotte, Mahé, Praslin, and Reunion Island) when exposed to three epidemic viruses: ZIKV, DENV-1, and CHIKV. All mosquito lines were found to be competent for the DENV-1 and CHIKV, with varying levels of susceptibility depending on both the virus and the mosquito line. In contrast, none of the lines were competent for the ZIKV strain tested. In general, vector competence parameters were higher for CHIKV than for DENV-1. This difference may be attributed not only to intrinsic genetic differences between the viruses, but also to the viral titers used in infectious blood meals. Indeed, a lower titer was used for the ZIKV strain (10^6^ PFU/mL) compared with the CHIKV strain (10^8^ PFU/mL), and the influence of bloodmeal titer on vector competence outcomes has been previously documented [29]. The differences observed between these viruses could also be related to the number of in vitro passages, as the DENV-1 and CHIKV used were at their fourth and tenth passages, respectively. An increased number of passages is known to promote genetic divergence from the original isolates and may lead to adaptive mutations that could influence virus–vector interactions [30–32]. Our analyses revealed that vector competence varied according to mosquito line and dpe, but not by the geographic origin of the mosquito lines (i.e., Comoros archipelago, Seychelles archipelago, and Reunion Island). The effect of mosquito lines on vector competence could be attributed to the number of generations of mosquito lines as there were differences in the duration of laboratory colonization (ranging from 1–9 generations for most lines to 31–37 generations for the *Ae. aegypti* line from Reunion Island) that could alter the mosquito genetic diversity [33, 34]. The influence of mosquito genetics on vector competence is well established [35]. Microsatellite-based genotyping has revealed high genetic diversity within *Ae. albopictus* populations from Reunion Island [36]. Extending such genetic analyses to *Ae. albopictus* and *Ae. aegypti* populations across the islands of the SWIO would provide valuable insights into the spatial structuring of genetic diversity in these species. These data could enable the identification of potential correlations between genetic variability and vector competence [37], while also contributing to a better understanding of the colonization history of these islands by *Ae. albopictus* and *Ae. aegypti* [15]. Regarding dpe, IR, DE, and TE increased over time, with significantly higher values observed in older mosquitoes. Specifically, for DENV-1, values were higher at 28 dpe compared with 21 and 14 dpe, and for CHIKV, values at 14 dpe were higher than at 7 dpe. These patterns are consistent with the replication kinetics of arboviruses within mosquitoes, progressing from initial infection of the midgut to systemic dissemination and subsequent release of infectious viral particles in the saliva [38].

The tested mosquito lines were not competent for the ZIKV strain PF-251013–18, which was isolated in French Polynesia and belongs to the Asian ZIKV lineage. This result is consistent with our previous study on *Ae. albopictus* and *Ae. aegypti* from Reunion Island, which showed no or very low TE after exposure to two others Asian ZIKV strains, namely MAS66 (from Malaysia) and PaRi_2015 (from Martinique) [23]. Similarly, *Ae. albopictus* from Reunion Island exposed to the Asian ZIKV strain NC-2014–5132 (from New Caledonia) did not show dissemination or transmission of the virus in the study of Vazeille et al. [39], whereas a TE of 10% was reported in the study of Bohers et al. [40]. Although variations in the vector competence of *Ae. albopictus* and *Ae. aegypti* for Asian ZIKV strains have been reported in other geographic regions [41–46], in the SWIO region, it appears that most of the tested *Aedes* lines were not competent or poorly competent for Asian ZIKV strains. This finding could help explain the absence of autochthonous transmission of ZIKV in the SWIO. However, ZIKV emergence could still occur in the region under favorable conditions, such as high mosquito densities [47] or the introduction of genetically distinct strains. In this context, cases of Asian Zika virus infections have recently been reported in France [48] and Italy [49] in travelers returning from the Seychelles, suggesting a potential low-level circulation. Moreover, the introduction of African ZIKV strains, for which high transmission rates by *Ae. albopictus* and *Ae. aegypti* from Reunion Island have been demonstrated [23], represents an additional risk. An epidemic caused by African ZIKV strains could be particularly concerning given their high pathogenic potential [50].

All tested *Ae. albopictus* and *Ae. aegypti* lines were competent to the local DENV-1 strain tested. These findings are consistent with our previous study on *Ae. albopictus* and *Ae. aegypti* from Reunion exposed to the same viral strain [24]. For *Ae. albopictus*, similar results have been reported for lines from diverse geographical origins exposed to other DENV-1 strains [51–53]. For *Ae. aegypti*, however, vector competence results are more variable at the global scale, as highlighted in the review by Souza-Neto et al. [8], which reported highly disparate TEs. For instance, no transmission was observed in *Ae. aegypti* from Key West, Florida [51], whereas lines from New Caledonia showed low transmission levels (TEs ranging from 3.0% to 13.0% at 14 dpe and from 0.0% to 13.0% at 21 dpe) [54]. In contrast, *Ae. aegypti* from Cuba exhibited higher TEs (25.0% at 14 dpe) [55]. Such variability in vector competence for DENV likely reflects complex interactions between mosquito genetics, viral strains, environmental factors, and experimental protocols [8, 52, 56]. Overall, the vector competence of *Ae. albopictus* and *Ae. aegypti* lines for DENV-1 observed in the present study aligns with the documented circulation of DENV-1 in the SWIO region [16]. Although the observed TEs were relatively low, the high mosquito densities on the islands could contribute to the occurrence of dengue in the SWIO. Dengue virus has four serotypes (DENV-1, DENV-2, DENV-3, and DENV-4), each with multiple genetic strains [57]. In the SWIO, all four serotypes have been reported in autochthonous and/or imported cases; however, only DENV-1 and DENV-2 have caused large-scale outbreaks in the region [16]. As vector competence studies have so far focused on DENV-1 and DENV-2 [24, 58, 59], future investigations should include DENV-3 and DENV-4 serotypes to see whether low-level circulation of DENV-3 and DENV-4 is explained by opportunity, with small numbers of imported cases from areas where these viruses are endemic, or by the reduced ability of local vectors to transmit these viruses.

When exposed to CHIKV, all *Ae. albopictus* and *Ae. aegypti* lines were competent and showed high TE, which is consistent with the various virus outbreaks reported in the SWIO [60]. Although differences were observed between mosquito lines, our findings align with previous studies conducted in the region on *Ae. albopictus* and *Ae. aegypti* lines from Reunion and Mayotte [58, 61, 62]. Similarly, our results are consistent with vector competence studies on Italian *Ae. albopictus* and American *Ae. albopictus* and *Ae. aegypti* lines exposed to the same CHIKV strain [22, 63]. This CHIKV strain carries the adaptive E1-226 V substitution, which has been associated with increased transmission by *Ae. albopictus* [6, 61]. However, in our study, the highest TE has been observed with the *Ae. aegypti* line AG_TBassin compared with *Ae. albopictus* lines. This could be explained by *Ae. albopictus* lines, as in the study of Vazeille et al. [35], where a lower transmission of CHIKV strains carrying E1-226 V was also reported in *Ae. albopictus* from Congo, supporting the role of mosquito genetics in vector competence [35]. Another hypothesis relates to the *Ae. aegypti* AG_TBassin line. Indeed, this line has been maintained in the laboratory since 2014 and reared for 31–37 generations, whereas the other mosquito lines were established in 2019 and reared for 1–9 generations. Long-term lab rearing for several generations could alter the vector competence parameters, notably through genetic bottleneck (reduced genetic diversity) and microbiome modification [24, 33, 64].

Finally, the study has several limitations. These include the high number of generations for mosquito lines, the high number of viral passages, and the small sample sizes in certain experimental trials. In some cases, only a few mosquitoes were available, and not all dpe were represented for each virus–mosquito line combination, which may have reduced the statistical power of the analyses. In addition, only two *Ae. aegypti* lines were used, compared with eight lines for *Ae. albopictus*. To enhance the robustness and generalizability of the findings, future investigations should be performed with wild-derived mosquito populations, low-passage viral strains, a larger sample sizes, and more *Ae. aegypti* lines.

## Conclusions

Our study demonstrates that the tested *Ae. albopictus* and *Ae. aegypti* lines from five islands in the SWIO were experimentally not competent vectors for the Asian ZIKV strain PF-251013–18, while they were competent for the DENV-1 and CHIKV strains tested. The vector competence parameters for DENV-1 and CHIKV appeared to be influenced by the mosquito lines and dpe, but not by the geographic origin of the mosquito lines. These findings might partly explain the current epidemiology of the three arboviruses in the SWIO, highlighting the importance of assessing mosquito vector competence. Such experimental approaches can contribute to a better evaluation of the risk of emergence or re-emergence of arboviruses associated with mosquito populations in the SWIO region. In this context, 20 years after the first outbreaks, the islands of the SWIO (Reunion and Mayotte) are currently experiencing another CHIKV outbreak. It would be therefore valuable to examine the vector competence of *Ae. albopictus* and *Ae. aegypti* from the different islands when infected with the viral strains presently circulating in the region.

## Supplementary Information

Additional file 1: Information on mosquito lines used for the vector competence experimentations with CHIKV, DENV and ZIKV. *NA* = *not available.*

Additional file 2: Vector competence details of *Aedes albopictus* and *Aedes aegypti* mosquitoes from SWIO exposed to the ZIKV strain. Infection rates (IR), dissemination efficiencies (DE), and transmission efficiencies (TE) are presented for 7, 14, and 21 days post-exposure (dpe) to infectious blood meals. IR = number of infected bodies among examined mosquitoes (%); DE = number of infected heads among examined mosquitoes (%); TE = number of infected saliva among examined mosquitoes (%). The fraction in parentheses represents the number of positive samples out of the total number of samples tested. The interval in brackets represents the 95% confidence interval of the value.* NA* = *not available.*

Additional file 3: Vector competence details of *Aedes albopictus* and *Aedes aegypti* mosquitoes from SWIO exposed to the DENV-1 strain. Infection rates (IR), dissemination efficiencies (DE), and transmission efficiencies (TE) are presented for 14, 21, and 28 days post-exposure (dpe) to infectious blood meals. IR = number of infected bodies among examined mosquitoes (%); DE = number of infected heads among examined mosquitoes (%); TE = number of infected saliva among examined mosquitoes (%); Mean/Median VT = mean or median of the viral titers found in saliva of the positive sample (log_10_ PFU/saliva). The fraction in parentheses represents the number of positive samples out of the total number of samples tested. The interval in brackets represents the 95% confidence interval of the value, or the first and third quartiles for the median. *NA* = *not available.*

Additional file 4: Results of post hoc analysis of estimated marginal means (emmeans) for comparison between modalities of the explanatory variables retained by the GLM for the DENV-1 strain tested. Comparisons were made using Tukey’s method on the estimated marginal means of the vector competence parameter for each modality, with their 95% confidence intervals indicated in brackets. The true mean of each modality and its 95% confidence interval in brackets are also given for information, with the fraction in parentheses representing the number of positive samples out of the total number of samples tested. The different explanatory variables, the three vector competence parameters (IR, DE or TE) and the two mosquito species were tested independently. Only comparisons with a significant difference (*P*-value < 0.05) are shown in the table. In red, significant explanatory variables that should not be considered due to the significance of their interactions. IR = infection rate; DE = dissemination efficiency; TE = transmission efficiency; dpe = post-exposure day; ML = mosquito line.

Additional file 5: Vector competence details of *Aedes albopictus* and *Aedes aegypti* mosquitoes from SWIO exposed to the CHIKV strain. Infection rates (IR), dissemination efficiencies (DE), and transmission efficiencies (TE) are presented for 7, and 14 days post-exposure (dpe) to infectious blood meal. IR = number of infected bodies among examined mosquitoes (%); DE = number of infected heads among examined mosquitoes (%); TE = number of infected saliva among examined mosquitoes (%); Mean/Median VT = mean or median of the viral titers found in saliva of the positive sample (log_10_ PFU/saliva). The fraction in parentheses represents the number of positive samples out of the total number of samples tested. The interval in brackets represents the 95% confidence interval of the value, or the first and third quartiles for the median. *NA* = *not available.*

Additional file 6: Results of post hoc analysis of estimated marginal means (emmeans) for comparison between modalities of the explanatory variables retained by the GLM for the CHIV strain tested. Comparisons were made using Tukey’s method on the estimated marginal means of the vector competence parameter for each modality, with their 95% confidence intervals indicated in brackets. The true mean of each modality and its 95% confidence interval in brackets are also given for information, with the fraction in parentheses representing the number of positive samples out of the total number of samples tested. The different explanatory variables, the three vector competence parameters (IR, DE or TE) and the two mosquito species were tested independently. Only comparisons with a significant difference (*P*-value < 0.05) are shown in the table. In red, significant explanatory variables that should not be considered due to the significance of their interactions. IR = infection rate; DE = dissemination efficiency; TE = transmission efficiency; dpe = post-exposure day; ML = mosquito line.

## Data Availability

All data generated or analyzed during this study are included in this published article.

## Abbreviations

AG: *Aedes aegypti* line
AL: *Aedes albopictus* line
ATP: Adenosine triphosphate
CHIKV: Chikungunya virus
DE: Dissemination efficiency
DENV: Dengue virus
DENV-1: Dengue virus serotype 1
dpe: Days post exposure
FBS: Fetal bovine serum
MEM: Eagle’s minimum essential medium
MOI: Multiplicity of infection
IR: Infection rate
PFU: Plaque forming unit
RH: Relative humidity
SWIO: Southwestern Indian Ocean
TE: Transmission efficiency
ZIKV: Zika virus
