# Supplementary material for: Vector competence of Aedes albopictus and Aedes aegypti from the islands of the Southwestern Indian Ocean for epidemic Zika, dengue, and chikungunya viruses
Source: Parasit Vectors. 2025 Dec 12;19:34. doi: 10.1186/s13071-025-07193-0 (PMC12817543; doi:10.1186/s13071-025-07193-0)
Supplement: Supplementary file 1 — Additional file 1: Information on mosquito lines used for the vector competence experimentations with CHIKV, DENV and ZIKV. NA = not available. [file 13071_2025_7193_MOESM1_ESM.pdf]

Island (geographic region)	Site	Mosquito species	Line code	Generation	Viruses tested	Number of mosquitoes tested			
						7 dpe	14 dpe	21 dpe	28 dpe
Mayotte (Comoros archipelago)	Combani	*Aedes albopictus*	AL_Combani	F4	*CHIKV*	**24**	**32**	*NA*	*NA*
				F5	*ZIKV*	**32**	**32**	**40**	*NA*
				F8	*DENV*	*NA*	*NA*	**24**	**19**
	Kawéni	*Aedes albopictus*	AL_Kaweni	F5	*CHIKV*	**32**	**32**	*NA*	*NA*
				F6	*ZIKV*	**32**	**32**	**40**	*NA*
				F9	*DENV*	*NA*	**24**	**32**	**39**
Grande Comore (Comoros archipelago)	Moroni	*Aedes albopictus*	AL_Moroni	F2	*CHIKV*	**26**	**35**	*NA*	*NA*
				F3	*ZIKV*	**32**	**32**	**40**	*NA*
				F7	*DENV*	*NA*	**32**	**48**	**48**
		Aedes aegypti	AG_Moroni	F2	*CHIKV*	**32**	**32**	*NA*	*NA*
				F3	*ZIKV*	**32**	**32**	**40**	*NA*
				F6	*DENV*	*NA*	**32**	**48**	**48**
Mahe (Seychelles archipelago)	Beauvallon	*Aedes albopictus*	AL_Beauvallon	F2	*CHIKV*	**24**	**35**	*NA*	*NA*
				F3	*ZIKV*	**32**	**24**	**40**	*NA*
				F6	*DENV*	*NA*	**32**	**47**	**40**
	Providence	*Aedes albopictus*	AL_Providence	F2	*CHIKV*	**24**	**27**	*NA*	*NA*
				F3	*ZIKV*	**24**	**32**	**22**	*NA*
				F6	*DENV*	*NA*	**32**	**48**	**48**
Praslin (Seychelles archipelago)	Praslin	*Aedes albopictus*	AL_Praslin	F3	*CHIKV*	**31**	*NA*	*NA*	*NA*
					ZIKV	*NA*	**32**	*NA*	*NA*
				F6	*DENV*	*NA*	**32**	**48**	**48**
Reunion Island (Mascarene archipelago)	Saint Gilles	*Aedes albopictus*	AL_Gilles	F1	*CHIKV*	**24**	**28**	*NA*	*NA*
					ZIKV	*NA*	*NA*	*NA*	*NA*
				F7	*DENV*	*NA*	**24**	**24**	**23**
	Saint Philippe	*Aedes albopictus*	AL_Philippe	F2	*CHIKV*	**32**	**36**	*NA*	*NA*
					ZIKV	*NA*	**32**	**31**	*NA*
				F5	*DENV*	*NA*	*NA*	**24**	*NA*
	Trois Bassins	*Aedes aegypti*	AG_TBassin	F31	*CHIKV*	**32**	**40**	*NA*	*NA*
					ZIKV	**32**	**32**	**40**	*NA*
				F37	*DENV*	*NA*	**32**	**48**	**30**
