# Supplementary material for: Vector competence of Aedes albopictus and Aedes aegypti from the islands of the Southwestern Indian Ocean for epidemic Zika, dengue, and chikungunya viruses
Source: Parasit Vectors. 2025 Dec 12;19:34. doi: 10.1186/s13071-025-07193-0 (PMC12817543; doi:10.1186/s13071-025-07193-0)
Supplement: Supplementary file 2 — Additional file 2: Vector competence details of Aedes albopictus and Aedes aegypti mosquitoes from SWIO exposed to the ZIKV strain. Infection rates (IR), dissemination efficiencies (DE), and transmission efficiencies (TE) are presented for 7, 14, and 21 days post-exposure (dpe) to infectious blood meals. IR = number of infected bodies among examined mosquitoes (%); DE = number of infected heads among examined mosquitoes (%); TE = number of infected saliva among examined mosquitoes (%). The fraction in parentheses represents the number of positive samples out of the total number of samples tested. The interval in brackets represents the 95% confidence interval of the value. NA = not available. [file 13071_2025_7193_MOESM2_ESM.pdf]

	7 dpe			14 dpe			21 dpe		
**Mosquito line**	**IR**	**DE**	**TE**	**IR**	**DE**	**TE**	**IR**	**DE**	**TE**
AL_Combani	0.0% (0/32) [0.0 – 10.7%]	0.0% (0/32) [0.0 – 10.7%]	0.0% (0/32) [0.0 – 10.7%]	0.0% (0/32) [0.0 – 10.7%]	0.0% (0/32) [0.0 – 10.7%]	0.0% (0/32) [0.0 – 10.7%]	0.0% (0/40) [0.0 – 8.8%]	0.0% (0/40) [0.0 – 8.8%]	0.0% (0/40) [0.0 – 8.8%]
AL_Kaweni	0.0% (0/32) [0.0 – 10.7%]	0.0% (0/32) [0.0 – 10.7%]	0.0% (0/32) [0.0 – 10.7%]	0.0% (0/32) [0.0 – 10.7%]	0.0% (0/32) [0.0 – 10.7%]	0.0% (0/32) [0.0 – 10.7%]	0.0% (0/40) [0.0 – 8.8%]	0.0% (0/40) [0.0 – 8.8%]	0.0% (0/40) [0.0 – 8.8%]
AL_Moroni	0.0% (0/32) [0.0 – 10.7%]	0.0% (0/32) [0.0 – 10.7%]	0.0% (0/32) [0.0 – 10.7%]	0.0% (0/32) [0.0 – 10.7%]	0.0% (0/32) [0.0 – 10.7%]	0.0% (0/32) [0.0 – 10.7%]	0.0% (0/40) [0.0 – 8.8%]	0.0% (0/40) [0.0 – 8.8%]	0.0% (0/40) [0.0 – 8.8%]
AG_Moroni	0.0% (0/32) [0.0 – 10.7%]	0.0% (0/32) [0.0 – 10.7%]	0.0% (0/32) [0.0 – 10.7%]	0.0% (0/32) [0.0 – 10.7%]	0.0% (0/32) [0.0 – 10.7%]	0.0% (0/32) [0.0 – 10.7%]	0.0% (0/40) [0.0 – 8.8%]	0.0% (0/40) [0.0 – 8.8%]	0.0% (0/40) [0.0 – 8.8%]
AL_Beauvallon	0.0% (0/32) [0.0 – 10.7%]	0.0% (0/32) [0.0 – 10.7%]	0.0% (0/32) [0.0 – 10.7%]	0.0% (0/24) [0.0 – 13.8%]	0.0% (0/24) [0.0 – 13.8%]	0.0% (0/24) [0.0 – 13.8%]	0.0% (0/40) [0.0 – 8.8%]	0.0% (0/40) [0.0 – 8.8%]	0.0% (0/40) [0.0 – 8.8%]
AL_Praslin	*NA*	*NA*	*NA*	0.0% (0/32) [0.0 – 10.7%]	0.0% (0/32) [0.0 – 10.7%]	0.0% (0/32) [0.0 – 10.7%]	*NA*	*NA*	*NA*
AL_Providence	0.0% (0/24) [0.0 – 13.8%]	0.0% (0/24) [0.0 – 13.8%]	0.0% (0/24) [0.0 – 13.8%]	0.0% (0/32) [0.0 – 10.7%]	0.0% (0/32) [0.0 – 10.7%]	0.0% (0/32) [0.0 – 10.7%]	0.0% (0/22) [0.0 – 14.9%]	0.0% (0/22) [0.0 – 14.9%]	0.0% (0/22) [0.0 – 14.9%]
AL_Gilles	*NA*	*NA*	*NA*	*NA*	*NA*	*NA*	*NA*	*NA*	*NA*
AL_Philippe	*NA*	*NA*	*NA*	0.0% (0/32) [0.0 – 10.7%]	0.0% (0/32) [0.0 – 10.7%]	0.0% (0/32) [0.0 – 10.7%]	0.0% (0/31) [0.0 – 11.0%]	0.0% (0/31) [0.0 – 11.0%]	0.0% (0/31) [0.0 – 11.0%]
AG_TBassin	**28.1% (9/32) [15.6 – 45.4%]**	0.0% (0/32) [0.0 – 10.7%]	0.0% (0/32) [0.0 – 10.7%]	**3.1% (1/32) [0.6 – 15.7%]**	0.0% (0/32) [0.0 – 10.7%]	0.0% (0/32) [0.0 – 10.7%]	**5.0% (2/40) [1.4 – 16.5%]**	**5.0% (2/40) [1.4 – 16.5%]**	0.0% (0/40) [0.0 – 8.8%]
