# Supplementary material for: Vector competence of Aedes albopictus and Aedes aegypti from the islands of the Southwestern Indian Ocean for epidemic Zika, dengue, and chikungunya viruses
Source: Parasit Vectors. 2025 Dec 12;19:34. doi: 10.1186/s13071-025-07193-0 (PMC12817543; doi:10.1186/s13071-025-07193-0)
Supplement: Supplementary file 3 — Additional file 3: Vector competence details of Aedes albopictus and Aedes aegypti mosquitoes from SWIO exposed to the DENV-1 strain. Infection rates (IR), dissemination efficiencies (DE), and transmission efficiencies (TE) are presented for 14, 21, and 28 days post-exposure (dpe) to infectious blood meals. IR = number of infected bodies among examined mosquitoes (%); DE = number of infected heads among examined mosquitoes (%); TE = number of infected saliva among examined mosquitoes (%); Mean/Median VT = mean or median of the viral titers found in saliva of the positive sample (log10 PFU/saliva). The fraction in parentheses represents the number of positive samples out of the total number of samples tested. The interval in brackets represents the 95% confidence interval of the value, or the first and third quartiles for the median. NA = not available. [file 13071_2025_7193_MOESM3_ESM.pdf]

	14 dpe					21 dpe					28 dpe				
Mosquito line	IR	DE	TE	Mean VT (Log$_{10}$PFU/saliva)	Median VT (Log$_{10}$PFU/saliva)	IR	DE	TE	Mean VT (Log$_{10}$PFU/saliva)	Median VT (Log$_{10}$PFU/saliva)	IR	DE	TE	Mean VT (Log$_{10}$PFU/saliva)	Median VT (Log$_{10}$PFU/saliva)
AL_Combani	*NA*	*NA*	*NA*	*NA*	*NA*	83.3% (20/24) [64.1 – 93.3%]	83.3% (20/24) [64.1 – 93.3%]	20.8% (5/24) [9.2 – 40.5%]	2.54 [0.00 – 2.97]	2.30 [1.88 – 2.40]	63.2% (12/19) [41.0 – 80.9%]	42.1% (8/19) [23.1 – 63.7%]	10.5% (2/19) [2.9 – 31.4%]	1.70 [0.00 – 2.57]	1.70 [1.57 – 1.80]
AL_Kaweni	66.7% (16/24) [46.7 – 82.0%]	16.7% (4/24) [6.7 – 35.9%]	4.2% (1/24) [0.7 – 20.2%]	2.51 [NA]	2.51 [NA]	50.0% (16/32) [33,6 – 66.4%]	31.3% (10/32) [18.0 – 48.6%]	9.4% (3/32) [3.2 – 24.2%]	3.41 [0.00 – 3.99]	3.18 [2.97 – 3.57]	46.2% (18/39) [31.6 – 61.4%]	41.0% (16/39) [27.1 – 56.6%]	28.2% (11/39) [16.5 – 43.8%]	3.04. [2.32 – 3.30]	2.48 [2.30 – 3.23]
AL_Moroni	50.0% (16/32) [33.6 – 66.4%]	6.3% (2/32) [1.7 – 20.1%]	0.0% (0/32) [0.0 – 10.7%]	*NA*	*NA*	41.7% (20/48) [28.8 – 55.7%]	20.8% (10/48) [11.7 – 34.3%]	8.3% (4/48) [3.3 – 19.6%]	3.12 [0.00 – 3.60]	2.90 [2.00 – 3.30]	50.0% (24/48) [36.4 – 63.6%]	14.6% (7/48) [7.2 – 27.2%]	6.3% (3/48) [2.1 – 16.8%]	1.52 [0.00 – 1.84]	1.40 [1.40 – 1.57]
AG_Moroni	62.5% (20/32) [45.3 – 77.1%]	6.3% (2/32) [1.7 – 20.1%]	3.1% (1/32) [0.6 – 15.7%]	1.70 [NA]	1.70 [NA]	50.0% (24/48) [36.4 – 63.6%]	29.2% (14/48) [18.2 – 43.2%]	2.1% (1/48) [0.4 – 10.9%]	1.40 [NA]	1.40 [NA]	79.2% (38/48) [65.7 – 88.3%]	70.8% (34/48) [56.8 – 81.8%]	18.8% (9/48) [10.2 – 31.9%]	3.08 [0.00 – 3.41	2.65 [2.00 – 3.23]
AL_Beauvallon	34.4% (11/32) [20.4 – 51.7%]	15.6% (5/32) [6.9 – 31.8%]	3.1% (1/32) [0.6 – 15.7%]	3.36 [NA]	3.36 [NA]	44.7% (21/47) [31.4 – 58.8%]	44.7% (21/47) [31.4 – 58.8%]	23.4% (11/47) [13.6 – 37.2%]	3.23 [2.32 – 3.50]	2.18 [1.94 – 3.60]	55.0% (22/40) [39.8 – 69.3%]	32.5% (13/40) [20.1 – 48.0%]	27.5% (11/40) [16.1 – 42.8%]	3.36 [2.44 – 3.64]	3.10 [2.73 – 3.32]
AL_Praslin	40.6% (13/32) [25.5 – 57.8%]	0.0% (0/32) [0.0 – 10.7%]	0.0% (0/32) [0.0 – 10.7%]	*NA*	*NA*	41.7% (20/48) [28.8 – 55.7%]	27.1% (13/48) [16.6 – 41.0%]	8.3% (4/48) [3.3 – 19.6%]	2.94 [0.00 – 3.51]	2.21 [2.10 – 2.95]	62.5% (30/48) [48.4 – 74.8%]	35.4% (17/48) [23.4 – 49.6%]	16.7% (8/48) [8.7 – 29.6%]	2.20 [0.00 – 2.54]	1.70 [1.40 – 2.21]
AL_Providence	31.3% (10/32) [18.0 – 48.6%]	3.1% (1/32) [0.6 – 15.7%]	0.0% (0/32) [0.0 – 10.7%]	*NA*	*NA*	27.1% (13/48) [16.6 – 41.0%]	22.9% (11/48) [13.3 – 36.5%]	8.3% (4/48) [3.3 – 19.6%]	2.69 [0.00 – 3.13]	2.46 [2.33 – 2.75]	60.4% (29/48) [46.3 – 73.0%]	39.6% (19/48) [27.0 – 53.7%]	35.4% (17/48) [23.4 – 49.6%]	3.23 [0.00 – 3.53]	2.51 [1.88 – 3.19]
AL_Gilles	62.5% (15/24) [42.7 – 78.8%]	8.3% (2/24) [2.3 – 25.8%]	0.0% (0/24) [0.0 – 13.8%]	*NA*	*NA*	50.0% (12/24) [31.4 – 68.6%]	29.2% (7/24) [14.9 – 49.2%]	8.3% (2/24) [2.3 – 25.8%]	1.70 [NA]	1.70 [NA]	78.3% (18/23) [58.1 – 90.3%]	56.5% (13/23) [36.8 – 74.4%]	34.8% (8/23) [19.8 – 55.1%]	2.12 [0.00 – 2.47]	1.57 [1.40 – 2.14]
AL_Philippe	*NA*	*NA*	*NA*	*NA*	*NA*	54.2% (13/24) [35.1 – 72.1%]	12.5% (3/24) [4.3 – 31.0%]	8.3% (2/24) [2.3 – 25.8%]	3.70 [0.00 – 4.83]	3.70 [3.40 – 3.88]	*NA*	*NA*	*NA*	*NA*	*NA*
AG_TBassin	62.5% (20/32) [45.3 – 77.1%]	6.3% (2/32) [1.7 – 20.1%]	0.0% (0/32) [0.0 – 10.7%]	*NA*	*NA*	50.0% (24/48) [36.4 – 63.6%]	39.6% (19/48) [27.0 – 53.7%]	14.6% (7/48) [7.2 – 27.2%]	2.50 [0.00 – 2.90]	1.78 [1.30 – 2.52]	66.7% (20/30) [48.8 – 80.8%]	53.3% (16/30) [36.1 – 69.8%]	16.7% (5/30) [7.3 – 33.6%]	2.18 [0.04 – 2.48]	2.24 [1.40 – 2.40]
