# Supplementary material for: Vector competence of Aedes albopictus and Aedes aegypti from the islands of the Southwestern Indian Ocean for epidemic Zika, dengue, and chikungunya viruses
Source: Parasit Vectors. 2025 Dec 12;19:34. doi: 10.1186/s13071-025-07193-0 (PMC12817543; doi:10.1186/s13071-025-07193-0)
Supplement: Supplementary file 4 — Additional file 4: Results of post hoc analysis of estimated marginal means (emmeans) for comparison between modalities of the explanatory variables retained by the GLM for the DENV-1 strain tested. Comparisons were made using Tukey’s method on the estimated marginal means of the vector competence parameter for each modality, with their 95% confidence intervals indicated in brackets. The true mean of each modality and its 95% confidence interval in brackets are also given for information, with the fraction in parentheses representing the number of positive samples out of the total number of samples tested. The different explanatory variables, the three vector competence parameters (IR, DE or TE) and the two mosquito species were tested independently. Only comparisons with a significant difference (P-value < 0.05) are shown in the table. In red, significant explanatory variables that should not be considered due to the significance of their interactions. IR = infection rate; DE = dissemination efficiency; TE = transmission efficiency; dpe = post-exposure day; ML = mosquito line. [file 13071_2025_7193_MOESM4_ESM.pdf]

Mosquito species	Vector competence parameters	Explanatory variables	Modality 1			Modality 2			*p-value* **<0.05 Emmeans post-hoc analysis**
			Identifier	Vector competence true mean	Vector competence estimated marginal mean	Identifier	Vector competence true mean	Vector competence estimated marginal mean	
Aedes albopictus	IR	dpe	21 dpe	45.8% [40.2 - 51.5%] (135/295)	48.7% [42.7 - 54.7%]	**28 dpe**	**57.8% [51.7 - 63.5%] (153/265)**	**62.0% [55.3 - 68.3%]**	0.0063
		ML	**AL_Combani**	**74.4% [59.8 - 85.1%] (32/43)**	**74.3% [59.0 - 85.4%]**	AL_Beauvallon	45.4% [36.7 - 54.3%] (54/119)	45.5% [36.7 - 54.5%]	0.0401
						AL_Providence	40.6% [32.5 - 49.3%] (52/128)	40.2% [32.0 - 49.0%]	0.0061
			**AL_Gilles**	**63.4% [51.8 - 73.6%] (45/71)**	**63.7% [51.9 - 74.0%]**				0.0387
	DE	dpe	-	-	-	-	-	-	-
		ML	-	-	-	-	-	-	-
		dpe | ML	14 dpe | AL_Beauvallon	15.6% [6.9 - 31.8%] (5/32)	15.6% [6.7 - 32.5%]	**21 dpe | AL_Beauvallon**	**44.7% [31.4 - 58.8] (21/47)**	**44.7% [31.3 - 58.9%]**	0.0259
			**21 dpe | AL_Combani**	**83.3% [64.2 - 93.3%] (20/24)**	**83.3% [63.1 - 93.6%]**	28 dpe | AL_Combani	42.1% [23.1 - 63.7%] (8/19)	42.1% [22.6 - 64.4%]	0.0073
			14 dpe | AL_Gilles	8.3% [2.3 - 25.9] (2/24)	8.3% [2.09 - 27.9%]	**28 dpe | AL_Gilles**	**56.5% [36.8 - 74.4%] (13/23)**	**56.5% [36.3 - 74.8%]**	0.0050
			14 dpe | AL_Providence	3.1% [0.6 - 15.7%] (1/32)	3.1% [0.4 - 19.1%]	**28 dpe | AL_Providence**	**39.6% [27.0 - 53.7%] (19/48)**	**39.6% [26.9 - 53.9%]**	0.0123
		ML | dpe	**AL_Combani | 21 dpe**	**83.3% [64.2 - 93.3%] (20/24)**	**83.3% [63.1 - 93.6%]**	AL_Providence | 21 dpe	22.9% [13.3 - 36.5%] (11/48)	22.9% [13.2 - 36.8%]	0.0003
						AL_Praslin | 21 dpe	27.1% [16.6 - 41.0%] (13/48)	27.1% [16.4 - 41.2%]	0.0012
						AL_Moroni | 21 dpe	20.8% [11.7 - 34.3%] (10/48)	20.8% [11.6 - 34.6%]	0.0002
						AL_Kaweni | 21 dpe	31.3% [18.0 - 48.6%] (10/32)	31.3% [17.7 - 49.0%]	0.0079
						AL_Gilles | 21 dpe	29.2% [14.9 - 49.2%] (7/24)	29.2% [14.6 - 49.8%]	0.0101
						AL_Philippe | 21 dpe	12.5% [4.3 - 31.0%] (3/24)	12.5% [4.1 - 32.4%]	0.0004
			**AL_Gilles | 28 dpe**	**56.5% [36.8 - 74.4%] (13/23)**	**56.5% [36.3 - 74.8%]**	AL_Moroni | 28 dpe	14.6% [7.3 - 27.2%] (7/48)	14.6% [7.1 - 27.6%]	0.0097
	TE	dpe	14 dpe	1.1% [0.3 - 4.1%] (2/176)	1.0% [0.2 - 3.9%]	**21 dpe**	**11.9% [8.7- 16.1%] (35/295)**	**11.1% [7.9 - 15.4%]**	0.0017
						**28 dpe**	**22.6% [18.0 - 28.1%] (60/265)**	**21.4% [16.0 - 28.0%]**	<0.0001
			21 dpe	11.9% [8.7 - 16.1%] (35/295)	11.1% [7.9 - 15.4%]				0.0034
		ML	**AL_Beauvallon**	**19.3% [13.2 - 27.3%] (23/119)**	**11.5% [6.4 - 19.8%]**	AL_Moroni	5.5% [2.7 - 10.9%] (7/128)	2.7% [1.1 - 6.3%]	0.0174

Aedes aegypti	**IR**	**dpe**	21 dpe	50.0% [40.2- 59.8%] (48/96)	50.0% [40.1 - 59.9%]	**28 dpe**	**74.4% [63.7- 82.7%] (58/78)**	**74.4% [63.6 - 82.8%]**	0.0036
	**DE**	**dpe**	14 dpe	6.3% [2.5- 15.0%] (4/64)	6.3% [2.4 - 15.5%]	**21 dpe**	**34.4% [25.6- 44.3%] (33/96)**	**34.4% [25.6 - 44.4%]**	0.0007
						**28 dpe**	**64.1% [53.0- 73.9%] (50/78)**	**64.1% [52.9 - 73.9%]**	<0.0001
			21 dpe	34.4% [25.6- 44.3%] (33/96)	34.4% [25.6 - 44.4%]				0.0004
	**TE**	**dpe**	-	-	-	-	-	-	-
		**ML | dpe**	-	-	-	-	-	-	-
		**dpe | ML**	-	-	-	-	-	-	-
