# Supplementary material for: Vector competence of Aedes albopictus and Aedes aegypti from the islands of the Southwestern Indian Ocean for epidemic Zika, dengue, and chikungunya viruses
Source: Parasit Vectors. 2025 Dec 12;19:34. doi: 10.1186/s13071-025-07193-0 (PMC12817543; doi:10.1186/s13071-025-07193-0)
Supplement: Supplementary file 5 — Additional file 5: Vector competence details of Aedes albopictus and Aedes aegypti mosquitoes from SWIO exposed to the CHIKV strain. Infection rates (IR), dissemination efficiencies (DE), and transmission efficiencies (TE) are presented for 7, and 14 days post-exposure (dpe) to infectious blood meal. IR = number of infected bodies among examined mosquitoes (%); DE = number of infected heads among examined mosquitoes (%); TE = number of infected saliva among examined mosquitoes (%); Mean/Median VT = mean or median of the viral titers found in saliva of the positive sample (log10 PFU/saliva). The fraction in parentheses represents the number of positive samples out of the total number of samples tested. The interval in brackets represents the 95% confidence interval of the value, or the first and third quartiles for the median. NA = not available. [file 13071_2025_7193_MOESM5_ESM.pdf]

	7 dpe					14 dpe				
**Mosquito line**	**IR**	**DE**	**TE**	**Mean VT (Log$_{10}$ PFU/saliva)**	**Median VT (Log$_{10}$ PFU/saliva)**	**IR**	**DE**	**TE**	**Mean VT (Log$_{10}$ PFU/saliva)**	**Median VT (Log$_{10}$ PFU/saliva)**
AL_Combani	91.7% (22/24) [74.2 – 97.7%]	37.5% (9/24) [21.2 – 57.3%]	8.3% (2/24) [2.3 – 25.8%]	3.18 [0.00 – 3.90]	3.18 [3.10 – 3.24]	68.8% (22/32) [51.4 – 82.0%]	59.4% (19/32) [42.3 – 74.5%]	25.0% (8/32) [13.3 – 42.1%]	2.12 [0.20 – .2.42]	1.80 [1.40 – 2.26]
AL_Kaweni	75.0% (24/32) [57.9 – 86.7%]	9.4% (3/32) [3.2 – 24.2%]	0.0% (0/32) [0.0 – 10.7%]	*NA*	*NA*	81.3% (26/32) [64.7 – 91.1%]	50.0% (16/32) [33.6 – 66.4%]	12.5% (4/32) [5.0 – 28.1%]	2.59 [0.00 – 3.04]	2.44 [1.64 – 2.79]
AL_Moroni	84.6% (22/26) [66.5 – 93.8%]	30.8% (8/26) [16.5 – 50.0%]	11.5% (3/26) [4.0 – 29.0%]	3.37 [0.00 – 3.91]	3.00 [3.00 – 3.48]	77.1% (27/35) [61.0 – 87.9%]	54.3% (19/35) [38.2 – 69.5%]	20.0% (7/35) [10.0 – 35.9%]	2.88 [0.00 – 3.19]	2.54 [2.44 – 2.96]
AG_Moroni	90.6% (29/32) [75.8 – 96.8%]	53.1% (17/32) [36.4 – 69.1%]	9.4% (3/32) [3.2 – 24.2%]	3.99 [0.00 – 4.54]	3.78 [3.60 – 4.13]	93.8% (30/32) [79.9 – 98.3%]	81.3% (26/32) [64.7 – 91.1%]	34.4% (11/32) [20.4 – 51.7%]	1.95 [1.59 – 2.13]	1.70 [1.40 – 2.14]
AL_Beauvallon	79.2% (19/24) [59.5 – 90.8%]	45.8% (11/24) [27.9 – 64.9%]	16.7% (4/24) [6.7 – 35.9%]	3.52 [0.00 – 3.84]	3.60 [3.36 – 3.70]	97.1% (34/35) [85.5 – 99.5%]	80.0% (28/35) [64.1 – 90.0%]	11.4% (4/35) [4.5 – 26.0%]	2.26 [0.00 – 2.59]	2.24 [1.94 – 2.43]
AL_Praslin	96.8% (30/31) [83.8 – 99.4%]	45.2% (14/31) [29.2 – 62.2%]	16.1% (5/31) [7.1 – 32.6%]	2.02 [1.58 – 2.24]	2.10 [1.88 – 2.18]	*NA*	*NA*	*NA*	*NA*	*NA*
AL_Providence	95.8% (23/24) [79.8 – 99.3%]	54.2% (13/24) [35.1 – 72.1%]	16.7% (4/24) [6.7 – 35.9%]	3.83 [0.00 – 4.36]	3.30 [3.00 – 3.89]	81.5% (22/27) [63.3 – 91.8%]	66.7% (18/27) [47.8 – 81.4%]	40.7% (11/27) [24.5 – 59.3%]	3.57 [0.00 – 3.95]	2.10 [1.57 – 3.26]
AL_Gilles	66.7% (16/24) [46.7 – 82.0%]	33.3% (8/24) [18.0 – 53.3%]	16.7% (4/24) [6.7 – 35.9%]	3.74 [0.00 – 4.15]	3.65 [3.00 – 3.95]	89.3% (25/28) [72.8 – 96.3%]	75.0% (21/28) [56.6 – 87.3%]	42.9% (12/28) [26.5 – 60.9%]	3.34 [0.00 – 3.76]	2.53 [2.00 – 2.92]
AL_Philippe	93.8% (30/32) [79.9 – 98.3%]	50.0% (16/32) [33.6 – 66.4%]	12.5% (4/32) [5.0 – 28.1%]	3.72 [0.00 – 4.20]	3.40 [3.24 – 3.78]	69.4% (25/36) [53.1 – 82.0%]	41.7% (15/36) [27.1 – 57.8%]	19.4% (7/36) [9.8 – 35.0%]	2.97 [0.00 – 3.35]	2.80 [2.35 – 2.88]
AG_TBassin	100.0% (32/32) [89.3 – 100.0%]	87.5% (28/32) [71.9 – 95.0%]	62.5% (20/32) [45.3 – 77.1%]	3.83 [3.51 – 4.01]	3.57 [3.30 – 3.81]	97.5% (39/40) [87.1 – 99.6%]	92.5% (37/40) [80.1 – 97.4%]	57.5% (23/40) [42.2 – 71.5%]	2.81 [2.30 – 3.04]	2.54 [2.14 – 2.82]
