# Supplementary material for: Vector competence of Aedes albopictus and Aedes aegypti from the islands of the Southwestern Indian Ocean for epidemic Zika, dengue, and chikungunya viruses
Source: Parasit Vectors. 2025 Dec 12;19:34. doi: 10.1186/s13071-025-07193-0 (PMC12817543; doi:10.1186/s13071-025-07193-0)
Supplement: Supplementary file 6 — Additional file 6: Results of post hoc analysis of estimated marginal means (emmeans) for comparison between modalities of the explanatory variables retained by the GLM for the CHIV strain tested. Comparisons were made using Tukey’s method on the estimated marginal means of the vector competence parameter for each modality, with their 95% confidence intervals indicated in brackets. The true mean of each modality and its 95% confidence interval in brackets are also given for information, with the fraction in parentheses representing the number of positive samples out of the total number of samples tested. The different explanatory variables, the three vector competence parameters (IR, DE or TE) and the two mosquito species were tested independently. Only comparisons with a significant difference (P-value < 0.05) are shown in the table. In red, significant explanatory variables that should not be considered due to the significance of their interactions. IR = infection rate; DE = dissemination efficiency; TE = transmission efficiency; dpe = post-exposure day; ML = mosquito line. [file 13071_2025_7193_MOESM6_ESM.pdf]

<table>
<tr><th>Mosquito species</th><th>Vector competence parameters</th><th>Explanatory variables</th><th>Modality 1</th><th></th><th></th><th>Modality 2</th><th></th><th></th><th>p-value < 0.05 Emmeans post-hoc analysis</th></tr>
<tr><th></th><th></th><th></th><th>Identifier</th><th>Vector competence true mean</th><th>Vector competence estimated marginal mean</th><th>Identifier</th><th>Vector competence true mean</th><th>Vector competence estimated marginal mean</th><th></th></tr>
<tr><td>Aedes albopictus</td><td>IR</td><td>dpe | ML</td><td>7 dpe | AL_Philippe</td><td>93.8% [79.9- 98.3%] (30/32)</td><td>93.8% [78.2 - 98.4%]</td><td>14 dpe | AL_Philippe</td><td>69.4% [53.1- 82.0%] (33/96)</td><td>69.4% [52.8 - 82.2%]</td><td>0.0206</td></tr>
<tr><td></td><td></td><td>ML | dpe</td><td>-</td><td>-</td><td>-</td><td>-</td><td>-</td><td>-</td><td>-</td></tr>
<tr><td></td><td>DE</td><td>dpe</td><td>-</td><td>-</td><td>-</td><td>-</td><td>-</td><td>-</td><td>-</td></tr>
<tr><td></td><td></td><td>ML</td><td>AL_Beauvallon</td><td>66.1% [53.4 - 76.9%] (39/59)</td><td>64.8% [50.8 - 76.6%]</td><td>AL_Kaweni</td><td>29.7% [19.9- 41.8%] (19/64)</td><td>24.3% [13.9 - 39.0%]</td><td>0.0027</td></tr>
<tr><td></td><td></td><td></td><td>AL_Providence</td><td>60.8% [47.1 – 73.0%] (31/51)</td><td>60.6% [46.6 - 73.0%]</td><td></td><td></td><td></td><td>0.0104</td></tr>
<tr><td></td><td></td><td>dpe | ML</td><td>7 dpe | AL_Beauvallon</td><td>45.8% [27.9 - 64.9%] (11/24)</td><td>45.8% [27.5 - 65.4%]</td><td>14 dpe | AL_Beauvallon</td><td>80.0% [64.1- 90.0%] (28/35)</td><td>80.0% [63.6 - 90.2%]</td><td>0.0083</td></tr>
<tr><td></td><td></td><td></td><td>7 dpe | AL_Gilles</td><td>33.3% [18.0 - 53.3%] (8/24)</td><td>33.3% [17.6 - 53.9%]</td><td>14 dpe | AL_Gilles</td><td>75.0% [56.6 - 87.3%] (21/28)</td><td>75.0% [56.1 - 87.6%]</td><td>0.0036</td></tr>
<tr><td></td><td></td><td></td><td>7 dpe | AL_Kaweni</td><td>9.4% [3.2 - 24.2%] (3/32)</td><td>9.4% [3.1 - 25.4%]</td><td>14 dpe | AL_Kaweni</td><td>50.0% [33.6 - 66.4%] (16/32)</td><td>50.0% [33.3 - 66.7%]</td><td>0.0012</td></tr>
<tr><td></td><td></td><td>ML | dpe</td><td>AL_Providence | 7 dpe</td><td>54.2% [35.1 - 72.1%] (13/24)</td><td>54.2% [34.6 - 72.5%]</td><td>AL_Kaweni | 7 dpe</td><td>9.4% [3.2 - 24.2%] (3/32)</td><td>9.4% [3.1 - 25.4%]</td><td>0.0198</td></tr>
<tr><td></td><td></td><td></td><td>AL_Philippe | 7 dpe</td><td>50.0% [33.6 - 66.4%] (16/32)</td><td>50.0% [33.3 - 66.7%]</td><td></td><td></td><td></td><td>0.0270</td></tr>
<tr><td></td><td></td><td></td><td>AL_Beauvallon | 14 dpe</td><td>80.0% [64.1- 90.0%] (28/35)</td><td>80.0% [63.6 - 90.2%]</td><td>AL_Philippe | 14 dpe</td><td>41.7% [27.1 - 57.8%] (15/36)</td><td>41.7% [26.9 - 58.1%]</td><td>0.0245</td></tr>
<tr><td></td><td>TE</td><td>dpe</td><td>7 dpe</td><td>12.0% [8.3 – 17.0%] (26/217)</td><td>11.0% [7.5- 16.1%]</td><td>14 dpe</td><td>23.6% [18.5 - 29.5%] (53/225)</td><td>23.8% [18.0- 30.7%]</td><td>0.0013</td></tr>
<tr><td></td><td></td><td>ML</td><td>AL_Providence</td><td>29.4% [18.7 - 43.0%] (15/51)</td><td>28.0% [17.3 - 41.9%]</td><td>AL_Kaweni</td><td>6.3% [2.5 - 15.0%] (4/64)</td><td>5.7% [2.1 - 14.5%]</td><td>0.0471</td></tr>
<tr><td></td><td></td><td></td><td>AL_Gilles</td><td>30.8% [19.9 - 44.3%] (16/52)</td><td>29.2% [18.3 - 43.0%]</td><td></td><td></td><td></td><td>0.0332</td></tr>
<tr><td>Aedes aegypti</td><td>IR</td><td>-</td><td>-</td><td>-</td><td>-</td><td>-</td><td>-</td><td>-</td><td>-</td></tr>
<tr><td></td><td>DE</td><td>dpe</td><td>7 dpe</td><td>70.3% [58.3 - 80.1%] (45/64)</td><td>72.7% [59.7 - 82.8%]</td><td>14 dpe</td><td>87.5% [77.9 - 93.3%] (63/72)</td><td>88.8% [79.0 - 94.4%]</td><td>0.0196</td></tr>
<tr><td></td><td></td><td>ML</td><td>AG_Moroni</td><td>67.2% [55.0 - 77.4%] (43/64)</td><td>68.3% [55.5 - 78.8%]</td><td>AG_TBassins</td><td>90.3% [81.3 - 95.2%] (65/72)</td><td>90.8% [81.5 - 95.6%]</td><td>0.0019</td></tr>
<tr><td></td><td>TE</td><td>ML</td><td>AG_Moroni</td><td>21.9% [13.5 - 33.4%] (14/64)</td><td>18.9% [10.4 - 31.9%]</td><td>AG_TBassins</td><td>59.7% [48.2 - 70.3%] (43/72)</td><td>60.0% [48.3 - 70.7%]</td><td><0.0001</td></tr>
<tr><td></td><td></td><td>dpe | ML</td><td>7 dpe | AG_Moroni</td><td>9.4% [3.2 - 24.2%] (3/32)</td><td>9.4% [3.1 - 25.4%]</td><td>14 dpe | AG_Moroni</td><td>34.4% [20.4 - 51.7%] (11/32)</td><td>34.4% [20.2 - 52.1%]</td><td>0.0226</td></tr>
<tr><td></td><td></td><td>ML | dpe</td><td>AG_Moroni | 7 dpe</td><td>9.4% [3.24 - 24.22%] (3/32)</td><td>9.4% [3.1 - 25.4%]</td><td>AG_TBassins | 7 dpe</td><td>62.5% [45.3- 77.1%] (20/32)</td><td>62.5% [44.9 - 77.3%]</td><td>0.0001</td></tr>
</table>
